# NUDT21‐mediated Alternative Polyadenylation of CDK19 Reprograms Cholesterol Biosynthesis to Drive Colorectal Cancer Progression

**DOI:** 10.1002/advs.202518346

**Published:** 2025-11-19

**Authors:** Yeping Yu, Jixin Ma, Minwei Zhou, Xiaodong Gu, Yiming Zhou, Zhenyang Li, Tianyu Zhang, Wei Gong, Chuanxin Huang, Jianbin Xiang

**Affiliations:** ^1^ Department of General Surgery Huashan Hospital Fudan University Shanghai 200040 China; ^2^ Shanghai Institute of Immunology & Department of Immunology and Microbiology Key Laboratory of Cell Differentiation and Apoptosis of Chinese Ministry of Education Faculty of Basic Medicine Shanghai Jiao Tong University School of Medicine Shanghai 200025 China; ^3^ Laboratory of General Surgery and Department of General Surgery Xinhua Hospital affiliated with Shanghai Jiao Tong University School of Medicine No. 1665 Kongjiang Road Shanghai 200092 China

**Keywords:** alternative polyadenylation, CDK19, cholesterol biosynthesis, colorectal cancer, NUDT21

## Abstract

Alternative polyadenylation (APA) is critical for shaping transcriptome diversity by generating mRNA isoforms that differ in the length of their 3′untranslated region (3′UTR). APA dysregulation, a defined feature of tumorigenesis, remains poorly understood in colorectal cancer (CRC). Here, CRISPR/Cas9 screening identifies APA regulator NUDT21 as a key driver of CRC progression. NUDT21 is overexpressed in CRC tumors and correlates with poor prognosis in CRC patients. Genetic ablation of NUDT21 impairs CRC cell proliferation and triggers CD8^+^ T cell anti‐tumor response through disrupting cholesterol biosynthesis, leading to delayed tumor progression in syngeneic mouse models. Mechanistically, NUDT21 promotes the generation of cyclin‐dependent kinase 19 (CDK19) mRNA isoforms with long 3′UTR by directing usage of distal polyadenylation sites. The long 3′UTR facilitates the export of CDK19 transcripts to the cytoplasm and supports their efficient translation, with no impact on their stability. Truncation of the long 3′UTR of Cdk19 recapitulates cholesterol biosynthesis and proliferative impairments, as well as the enhanced anti‐tumor CD8^+^ T cell activity observed upon NUDT21 depletion, whereas CDK19 overexpression rescues these phenotypes. The findings establish the NUDT21‐CDK19 axis as a central hub integrating APA manner, cholesterol metabolic reprogramming, and immune evasion in CRC, unveiling potential therapeutic opportunities.

## Graphical abstract

NUDT21 is overexpressed in colorectal cancer tumors and promotes colorectal cancer progression. NUDT21 promotes the production of a cyclin‐dependent kinase 19 (CDK19) mRNA isoform with a long 3′ UTR, which enhances cytoplasmic export and translation. Truncation of the CDK19 long 3′ UTR phenocopies NUDT21 loss, recapitulating the defects in cholesterol biosynthesis and the activation of anti‐tumor CD8⁺ T cell immunity.

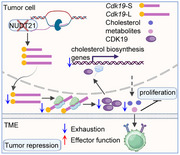

## 1 Introduction

Colorectal cancer (CRC) ranks as the third most prevalent cancer and the second leading cause of cancer‐related death worldwide, accounting for ≈10% of global cancer mortality.^[^ ^1^ ^]^ Despite advances in systemic therapies, the prognosis of patients with advanced or metastatic CRC remains unsatisfactory.^[^ ^2^, ^3^ ^]^ There is a continuing necessity for a comprehensive understanding of novel mechanisms driving CRC progression and identification of innovative therapeutic strategies.

Alternative polyadenylation (APA) is a widespread post‐transcriptional regulatory mechanism that generates messenger RNA (mRNA) isoforms with distinct 3′untranslated region (3′UTR) lengths through the selective usage of alternative polyadenylation (polyA) sites (PASs).^[^ ^4^ ^]^ APA is orchestrated by four core protein subcomplexes, including cleavage and polyadenylation factor (CPSF), cleavage stimulation factor (CSTF), and mammalian cleavage factors I and II (CFIm and CFIIm).^[^ ^5^ ^]^ The 3′UTR of mRNA harbors regulatory elements such as binding sites for microRNAs (miRNAs) and RNA‐binding proteins (RBPs), thereby modulating transcript stability, translation efficiency, nuclear export, and subcellular localization, independent of mRNA abundance or splicing.^[^ ^6^, ^7^ ^]^ Although tightly regulated in health conditions, dysregulated APA has been implicated in multiple human diseases, including cancer.^[^ ^8^ ^]^ Dysregulated APA arises through two primary mechanisms: mutations or germline variations that either create or destroy specific PASs, or altered expression of APA regulators.^[^ ^5^, ^9^, ^10^ ^]^ Global 3′UTR shortening frequently occurs in tumors, enabling oncogenes such as MYC and CCND1 to escape miRNA‐mediated repression.^[^ ^11^, ^12^ ^]^ Previous studies have linked APA patterns to clinical features of CRC, including patient prognosis and immune infiltration.^[^ ^13^, ^14^ ^]^ However, the molecular mechanisms driving APA reprogramming and aberrant APA events‐mediated tumor metabolism rewiring in CRC remain poorly elucidated.

In parallel, cholesterol metabolism has emerged as a hallmark of tumor biology.^[^ ^15^ ^]^ Cancer cells characteristically upregulate both de novo cholesterol biosynthesis and exogenous cholesterol uptake to sustain rapid proliferation, invasion capacity, and acquired resistance to therapy.^[^ ^16^, ^17^ ^]^ Within the tumor microenvironment (TME), cholesterol and its metabolites accumulate aberrantly and reshape the immune landscape, facilitating immune evasion and disease progression. For example, cholesterol within the TME promotes CD8⁺ T cell exhaustion by inducing the expression of immune checkpoints, such as PD‐1 and LAG‐3.^[^ ^18^ ^]^ Furthermore, tumor‐derived cholesterol metabolites can also activate myeloid‐derived suppressor cells (MDSCs) and skew tumor‐associated macrophages (TAMs) toward an immunosuppressive M2‐like phenotype, ultimately fostering immunosuppressive reprogramming.^[^ ^19^ ^]^ MDSCs and M2‐like TAMs further indirectly impair CD8⁺ T cell‐mediated anti‐tumor responses. Preclinical and clinical evidence suggest that targeting cholesterol metabolism suppresses tumor growth, reprograms the immune landscape, and restores T cell‐dependent immune surveillance.^[^ ^20^, ^21^ ^]^ Across diverse cancer types, genetic and epigenetic alterations rewire cholesterol metabolic pathways to promote tumorigenesis via TME modulation.^[^ ^22^, ^23^ ^]^ However, it remains unknown whether APA contributes to the activation of cholesterol biosynthesis programs in CRC.

In this study, we hypothesized that APA regulators might serve as molecular bridges connecting RNA regulation to metabolic and immunological reprogramming in CRC. Through a small‐scale CRISPR/Cas9 screening, we identified nudix hydrolase 21 (NUDT21), a core component of the CFIm complex (also known as CFIm25), as a critical driver of CRC progression. Integrative transcriptomic and PAS analysis revealed that NUDT21 promotes distal PAS (dPAS) usage and facilitates the generation of cyclin‐dependent kinase 19 (CDK19) mRNA isoforms with long 3′UTR. Functionally, the NUDT21‐CDK19 axis enhances cholesterol biosynthesis, thereby facilitating CRC cell proliferation and impairing anti‐tumoral CD8⁺ T cell responses. Collectively, these findings uncover an unrecognized mechanistic link between APA and metabolic regulation in CRC, and highlight the therapeutic potential of targeting RNA processing pathways for cancer treatment.

## 2 Results

### 2.1 CRISPR/Cas9 Screening Identifies the APA Regulator NUDT21 as a Key Mediator for CRC Progression

**Figure 1:**
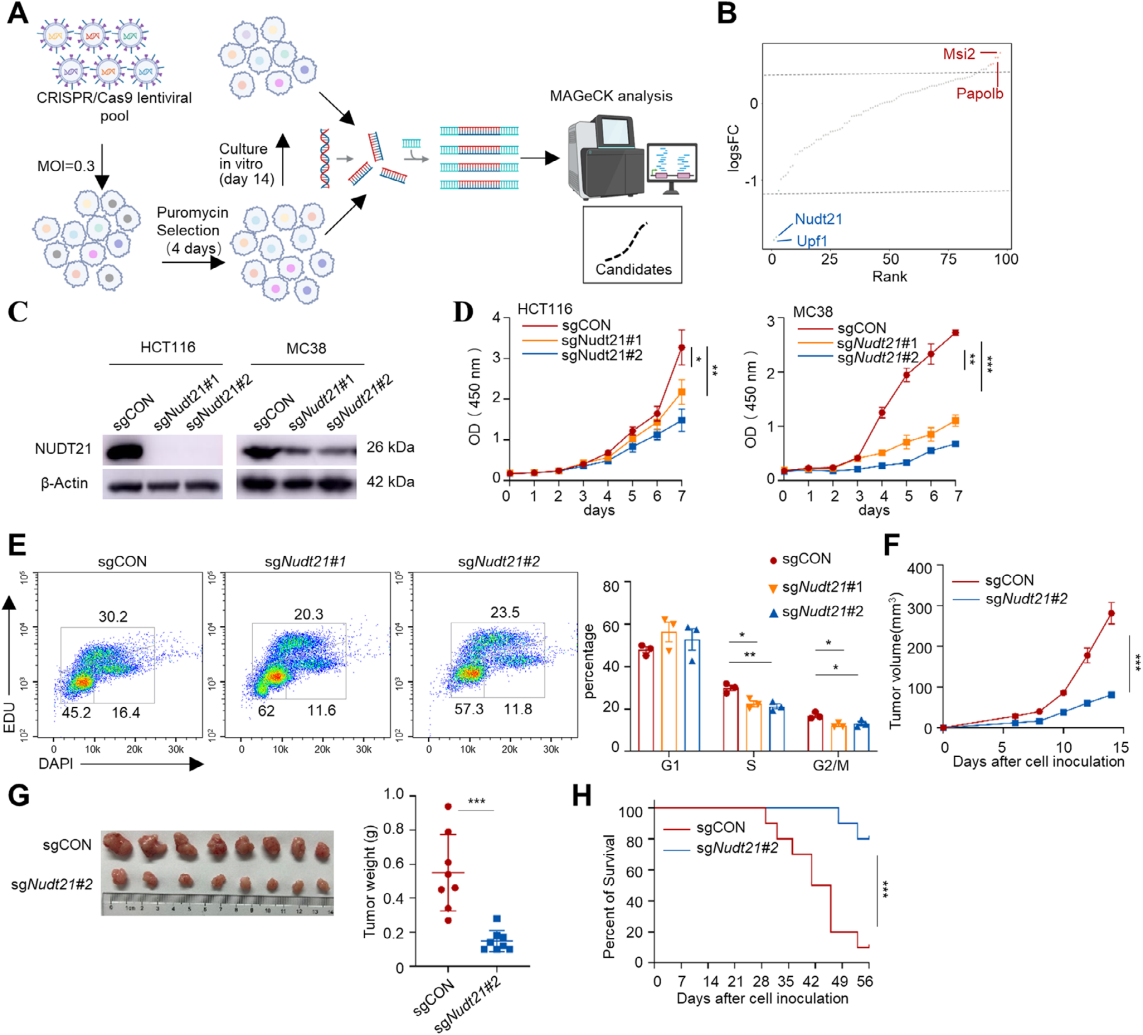
CRISPR screening identifies NUDT21 as a key mediator for CRC progression. A) Schematic diagram of in vitro CRISPR screening to identify potential targets involving in RNA regulation for CRC progression. B) MAGeCK analysis and ranked dot plots illustrating the top depleted (blue) or enriched genes (red) based on the screening data. C) Western blot showing the efficacy of sgRNA‐mediated Nudt21 knockout in HCT116 and MC38 cell lines. D) CCK‐8 analysis of HCT116 and MC38 cells expressing sgCON or sg*Nudt21* (n=5). E) Cell cycle analysis of sgCON or sg*Nudt21* MC38 cells (n=3). F, G) Growth curve, representative images, and weight quantification of subcutaneous sgCON or sg*Nudt21* MC38 tumors in C57BL/6J mice (n=8). H) Kaplan‐Meier plots of overall survival in mice inoculated subcutaneously with sgCON or sg*Nudt21* MC38 cells (n=10). Data are shown as mean ± SEM (D, E, F, G, and H). *p* values are determined by one‐way ANOVA (E) and two‐way ANOVA tests (D and F), Welch's *t* test (G), and the Log‐rank test (H). ns, no significance, ^*^ *p* < 0.05, ^**^ *p* < 0.01 and ^***^ *p* < 0.001.

To systematically uncover RNA regulators critical for CRC growth, we conducted an in vitro CRISPR/Cas9 screening in HCT116 cells using a lentiviral knockout library targeting 98 genes involved in RNA surveillance (including RNA splicing, APA, nucleocytoplasmic export, RNA degradation, and so on), with at least four single guide RNAs (sgRNAs) per gene (**Figure** **1**A). After puromycin selection for 4 days to enrich edited cells, populations were either harvested immediately (baseline control) or cultured for an additional two weeks. Genomic DNA was extracted from each group, and integrated sgRNAs were amplified and sequenced. MAGeCK analysis revealed a subset of sgRNAs progressively lost over time, with NUDT21 emerging as the top hit, pinpointing it as a key regulator of CRC growth (Figure 1B; Figure S1, Supporting Information).

To further address the functional contribution of NUDT21 to CRC growth, we generated *NUDT21*‐deficient human CRC cell lines, HCT116 and HT29, as well as murine MC38 cells, using CRISPR/Cas9‐mediated gene editing. To minimize off‐target effects, two independent sgRNAs were employed. Immunoblotting confirmed near‐complete loss of NUDT21 in HCT116 and HT29 introduced with *NUDT21*‐specific sgRNAs, whereas only partial depletion was achieved in MC38 cells, suggesting that full NUDT21 ablation may be lethal in this line (Figure 1C; Figure S2A, Supporting Information). NUDT21 ablation significantly suppressed cell proliferation and colony formation of HCT116 and HT29 cells (Figure 1D; Figure S2B,C, Supporting Information). Similar growth inhibition was observed in MC38 cells following *Nudt21* knockdown (Figure 1D; Figure S2B,C, Supporting Information). Cell cycle analysis revealed a reduced G_2_/M and S phase proportion in *Nudt21*‐knockdown MC38 cells (Figure 1E), indicating that Nudt21 promotes proliferation by facilitating cell cycle progression. However, *Nudt21* depletion did not affect cell apoptosis under in vitro conditions (Figure S2D, Supporting Information). To evaluate the effects of Nudt21 loss in vivo, control or *Nudt21*‐knockdown MC38 cells were subcutaneously implanted into female C57BL/6J mice. Nudt21 knockdown markedly suppressed tumor growth (Figure 1F,G) and extended the survival of MC38 tumor‐bearing mice compared with controls (Figure 1H). These findings identify NUDT21 as a key driver of CRC progression.

### 2.2 NUDT21 was Overexpressed in Human CRC Specimens and Correlated with Poor Patient Prognosis

**Figure 2:**
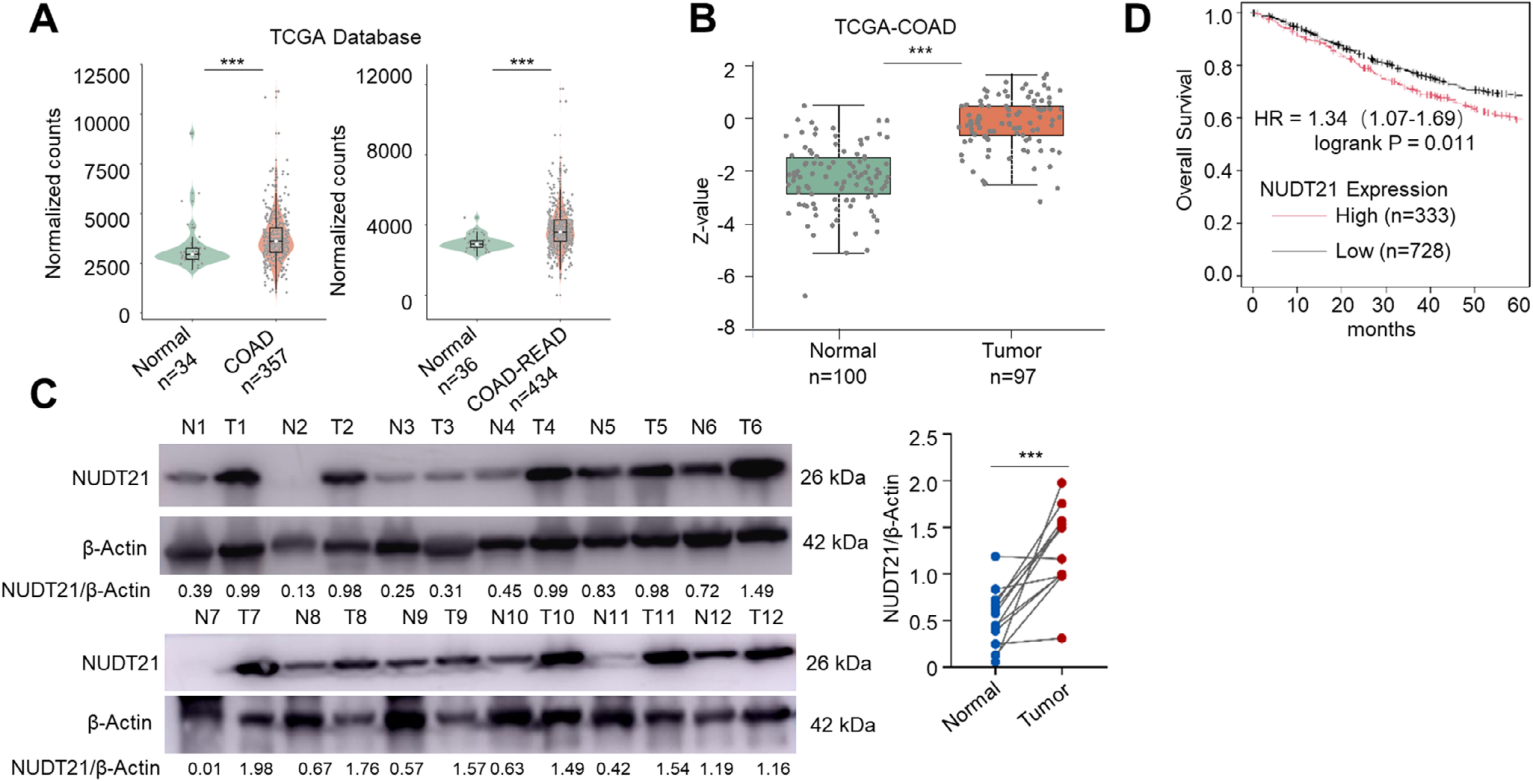
NUDT21 was overexpressed in human CRC specimens and correlated with poor patient prognosis. A) Boxplots showing the relative *NUDT21* mRNA levels in human colon cancer specimens and normal tissues from the TCGA database. COAD, colon adenocarcinoma; READ, rectal adenocarcinoma. B) Boxplots showing the relative NUDT21 protein levels in human colon cancer specimens and normal tissues by analyzing the CPTAC‐CRC proteomics dataset. C) Immunoblotting of NUDT21 protein in human CRC specimens and matched adjacent normal tissues. Quantification is shown on the right. D) Kaplan‐Meier curves of overall survival (n=1061) in patients from the TCGA‐COAD dataset according to *NUDT21* expression levels. *p* values are determined by the paired Student *t*’ test (C) and the Log‐rank test (D). ^***^ *p* < 0.001.

NUDT21 is downregulated in many human cancers, including glioblastoma and hepatocellular carcinoma, and mediates tumor suppression.^[^ ^24^, ^25^ ^]^ However, a recent study reported that NUDT21 expression is increased in gastric cancer, serving as an oncogene.^[^ ^26^ ^]^ To determine its relevance in CRC, we analyzed TCGA datasets and observed cancer type‐specific expression patterns of NUDT21, with its mRNA levels either upregulated or downregulated in cancer tissues compared to their normal controls (Figure S3A, Supporting Information). Notably, we observed significantly higher *NUDT21* mRNA levels in COAD and READ tissues compared with adjacent normal tissues (**Figure** **2**A). Correspondingly, NUDT21 protein abundance was also elevated in COAD, as shown in the TCGA proteomic dataset (Figure 2B). Our analysis further revealed significant genomic amplification of *NUDT21* in CRC tumor tissues compared with normal tissues, with no change in DNA methylation levels at its transcription start site (Figure S3B,C, Supporting Information), suggesting that copy‐number gains may drive its elevated expression. Analysis of 12 paired CRC specimens from Huashan Hospital confirmed higher NUDT21 protein abundance in tumors than in adjacent normal tissues (Figure 2C). Kaplan‐Meier survival analysis demonstrated that high NUDT21 expression was associated with worse overall survival in CRC patients (Figure 2D). Together, these findings indicate that NUDT21 is aberrantly overexpressed in CRC and its elevated expression is associated with poor clinical outcomes.

### 2.3 Nudt21 Deficiency Hinders CRC Growth by Disrupting Cholesterol Biosynthesis

**Figure 3:**
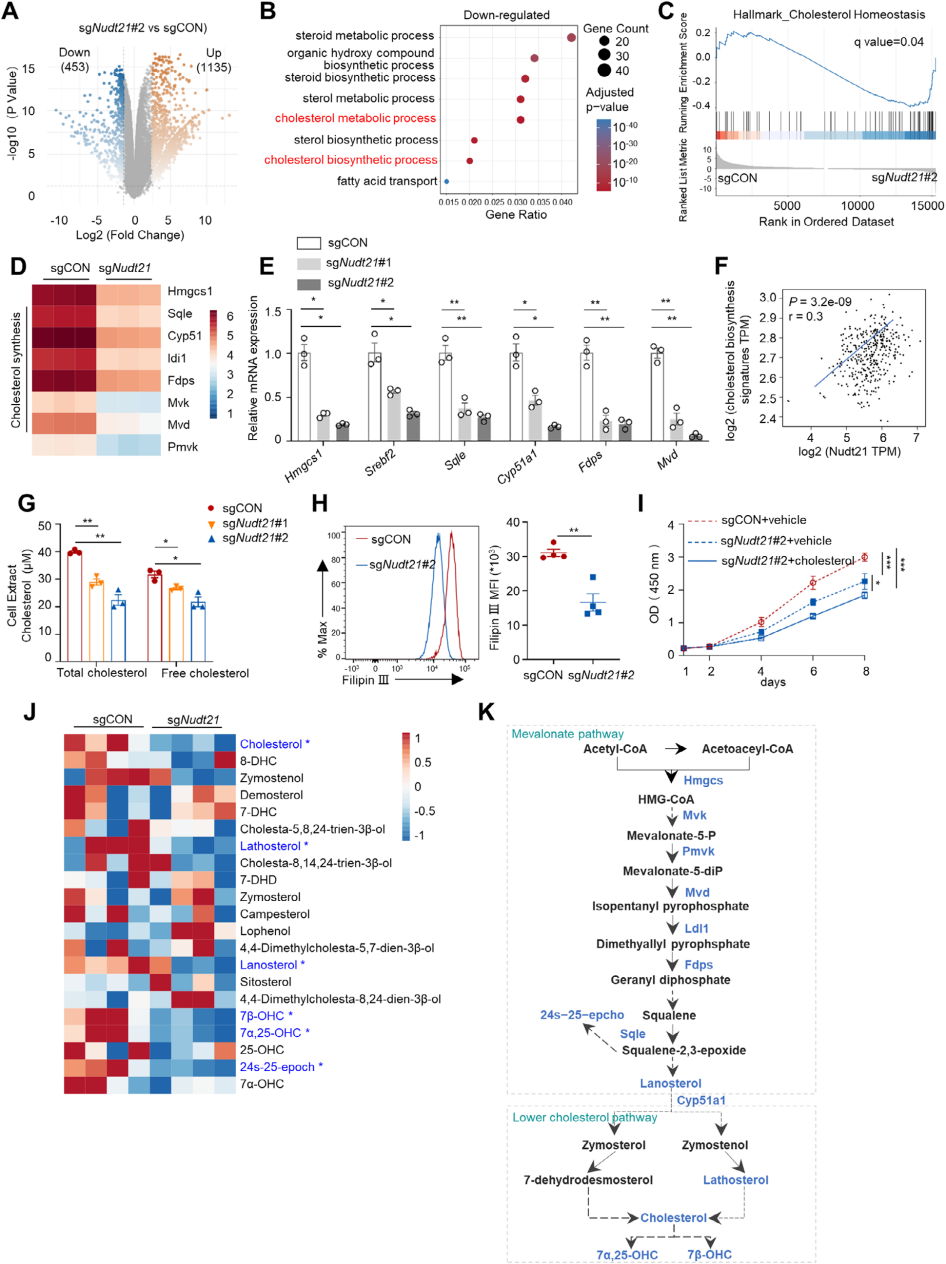
NUDT21 deficiency retards CRC growth by disrupting cholesterol biosynthesis. A) Volcano plots showing differentially expressed genes between sgCON and sg*Nudt21* MC38 cells (n=3). B) Go enrichment analysis of top enriched pathways involving downregulated gene sets in sg*Nudt21* MC38 cells, identified in (A) (n=3). C) GSEA analysis of cholesterol homeostasis gene signature based on downregulated genes in sg*Nudt21* MC38 cells (n=3). D) Heatmap displaying the relative expression of cholesterol biosynthesis‐related genes in sgCON and sg*Nudt21* MC38 cells (n=3). E) RT‐qPCR analysis of cholesterol biosynthesis‐related gene expression in sgCON and sg*Nudt21* MC38 cells (n=3). F) Pearson correlation between NUDT21 expression and the score of cholesterol biosynthesis‐related gene signature in TCGA‐CRC database (n=367). G) Boxplot of total cholesterol and free cholesterol concentrations in sgCON or sg*Nudt21* MC38 cells (n=3). H) FACS analysis of Filipin III staining of cholesterol in sgCON or sg*Nudt21* MC38 cells (n=3). I) CCK‐8 analysis of sgCON or sg*Nudt21* MC38 cells treated with or without 75 µm exogenous cholesterol (n=5). J) Heatmap showing concentrations of cholesterol and its metabolites in tumoral interstitial fluids from subcutaneous sgCON or sg*Nudt21* MC38 tumor tissues (n=4). K) Integrated schematic illustration of the cholesterol biosynthesis pathway and metabolites. The downregulated metabolites and genes in the context of *Nudt21* knockdown were marked in blue. Data are presented as the mean ± SEM (E and G). *p* values are determined by two‐way ANOVA (E, G, and I) or Welch's *t* test (H). ^*^ *p* < 0.05 ^**^ *p* < 0.01, ^***^ *p* < 0.001.

To elucidate the mechanisms underlying *Nudt21* loss‐mediated tumor suppression in CRC, we performed RNA sequencing of control and *Nudt21*‐knockdown MC38 cells. This analysis yielded 1588 differentially expressed genes (DEGs), with 71.5% (1135 genes) upregulated and 28.5% (453 genes) downregulated in *Nudt21*‐knockdown MC38 cells relative to controls (**Figure** **3**A). Gene Ontology (GO) analysis revealed that downregulated genes were significantly enriched in metabolic pathways, especially cholesterol biosynthesis, which ranked among the top ten enriched pathways, with Gene Set Enrichment Analysis (GSEA) data confirming depression of cholesterol homeostasis signatures (Figure 3B,C). In parallel, GO and GSEA analysis also indicated the activation of IFN‐γ response pathways in *Nudt21*‐knockdown cells, and RT‐qPCR verified several representative up‐regulated genes among this pathway (Figure S4A–D, Supporting Information). These results suggest that tumor‐intrinsic Nudt21 suppresses anti‐immune responses. Given the established role of cholesterol and its derivatives in tumor progression,^[^ ^15^ ^]^ we focused on the regulation of Nudt21 on cholesterol biosynthesis. According to RNA‐seq data, *Nudt21* knockdown reduced the expression of multiple key cholesterol biosynthetic genes, including *Hmgcs1, Sqle, Cyp51a1, Fdps*, and *Mvd* (Figure 3D), further validated by RT‐qPCR (Figure 3E). Notably, *Srebf2*, a master transcription activator of cholesterol and fatty acid synthesis, was also increased upon Nudt21 knockdown (Figure 3E). The positive correlation between *NUDT21* expression and the cholesterol synthetic gene signature (Srebf2, Sqle, Hmgcs1, Mvk, Mvd, Cyp51, Idi1, and Fdps) was also observed in TCGA datasets (Figure 3F). Consistent with these transcriptional changes, *Nudt21*‐deficient MC38 cells displayed reduced total and free cholesterol compared with control cells, as quantified by Amplex Red Cholesterol Assay (Figure 3G), with Filipin III staining providing orthogonal validation (Figure 3H). Moreover, exogenous cholesterol supplementation partially rescued the impaired growth of *Nudt21*‐deficient CRC cells (Figure 3I), indicating that Nudt21 supports tumor proliferation in part through sustaining cholesterol biosynthesis. Extending these findings in vivo, metabolomic profiling of tumor interstitial fluid exhibited decreases in cholesterol, its biosynthetic intermediates (lanosterol, lathosterol), and oxidative derivatives (oxysterols) in *Nudt21*‐knockdown tumors (Figure 3J). Collectively, these findings establish Nudt21 as a key regulator of cholesterol biosynthesis and metabolism in CRC, acting through transcriptional control of cholesterol biosynthetic genes (Figure 3K).

### 2.4 Nudt21 Knockdown in Tumor Cells Enhances Anti‐Tumor CD8^+^ T Cell Immunity

**Figure 4:**
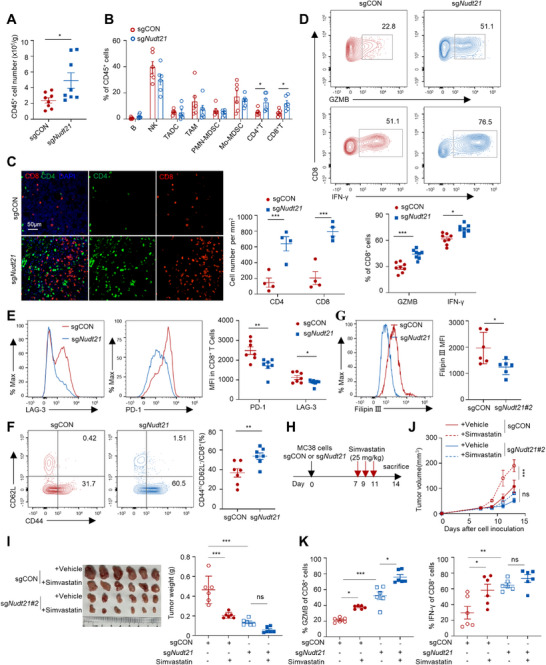
Nudt21 knockdown in tumor cells enhances anti‐tumor CD8^+^ T cell immunity. Female C57BL/6J mice were inoculated subcutaneously with 5×10^5^ sgCON or sg*Nudt21#2* MC38 cells. The TME was analyzed at day 14 post injection A–G). (A) FACS analysis of the numbers of CD45^+^ immune cells in sgCON or sg*Nudt21* MC38 tumors (n=8). (B) FACS analysis of tumor‐infiltrating immunocytes proportions in sgCON or sg*Nudt21* MC38 tumors (n=6). (C) Representative mIHC images of infiltrating CD4^+^ and CD8^+^ T cells in sgCON or sg*Nudt21* MC38 tumors and quantifications (n=4). Scale bar, 50 µm. (D) FACS analysis of GZMB and IFN‐γ production in tumor‐infiltrating CD8^+^ T cells (n=8). (E) FACS analysis of the surface PD‐1 and LAG‐3 expression in tumor‐infiltrating CD8^+^ T cells (n=8). (F) FACS analysis of CD62L and CD44 expression in tumor‐infiltrating CD8^+^ T cells (n=8). (G) FACS analysis of Filipin III staining of tumor cells and quantifications (n=6). H–K) The effect of simvastatin on subcutaneous sgCON or sg*Nudt21#2* MC38 tumors (n=5). Schematic of the simvastatin treatment schedule (H). Representative images and quantification of tumor weights (I), growth curve (J), and GZMB‐ and IFN‐γ‐ producing CD8^+^ T cells (K) of sgCON or sg*Nudt21* MC38 tumors treated with or without simvastatin (n=6). Each symbol represents an individual mouse. All data are shown as mean ± SEM. Statistical analysis was performed using Welch's *t* test (A, B, C, D, E, F, and G) or two‐way ANOVA (I, J, and K). ^*^ *p* < 0.05, ^**^ *p* < 0.01, ^***^ *p* < 0.001.

Tumor‐derived cholesterol and its metabolites are known to reshape TME to promote tumor progression.^[^ ^18^, ^27^ ^]^ We sought to assess whether Nudt21 deficiency‐mediated suppression of cholesterol biosynthesis enhances anti‐tumor responses. To this end, tumor‐infiltrating immunocytes were isolated from *Nudt21*‐knockdown and control tumors and subjected to cytometry analysis. Flow cytometry demonstrated a significant increase in CD45⁺ immunocytes infiltration in *Nudt21*‐knockdown tumors (**Figure** **4**A). Moreover, the proportions of both CD8⁺ and CD4⁺ T cell populations from these tumors were significantly increased compared with control tumors (Figure 4B). mIHC staining further confirmed elevated infiltration of both T cell subsets (Figure 4C). Given the pivotal role of CD8⁺ T cells in anti‐tumor immunity, we examined their functional state in *Nudt21*‐deficient tumors. Downregulated tumor‐intrinsic Nudt21 increased the frequencies of tumor‐infiltrating GranzymeB (GZMB)^+^ and Interferon Gamma (IFN‐γ)^+^ CD8^+^ T cells (Figure 4D), and reduced the expression of exhaustion markers PD‐1 and LAG‐3 (Figure 4E), compared with control counterparts. Moreover, Nudt21 deficiency not only increased overall CD8⁺T cell infiltration but also favored the expansion of effector memory/effector (CD44^hi^CD62L^−^) CD8⁺ T cells (Figure 4F). These findings suggest that tumor‐derived Nudt21 restrains antitumor CD8^+^ T cell immunity. Consistent with our earlier findings of reduced cholesterol in *Nudt21*‐deficient tumors (Figure 3J), Filipin III staining confirmed diminished cellular cholesterol levels in *Nudt21*‐deficient tumor cells (Figure 4G). Tumor‐derived cholesterol has been reported to directly suppress CD8^+^ T cell antitumor immune responses within the TME.^[^ ^18^ ^]^ We found that *Nudt21*‐deficient tumor interstitial fluid induced higher expression of PD‐1 and LAG‐3 on CD8^+^ T cells cultured in vitro than control (Figure S5B, Supporting Information). Methyl‐β‐cyclodextrin‐mediated cholesterol depletion largely abrogated these differential effects (Figure S5B, Supporting Information), indicating that *Nudt21* deficiency enhances antitumor CD8^+^ T cell immunity, partially through cholesterol reduction. To directly assess whether diminished cholesterol contributes to impaired tumor growth in *Nudt21*‐deficient tumors, we employed pharmacological inhibition with simvastatin, an HMG‐CoA reductase (HMGCR) reductase inhibitor. In line with previous observations,^[^ ^28^ ^]^ simvastatin robustly suppressed the tumor growth and activated CD8⁺ T cell responses in sgCON MC38 tumors (Figure 4H–K). In contrast, the effect of simvastatin on *Nudt21*‐knockdown tumors was modest and failed to reach statistical significance, further demonstrating the involvement of cholesterol in tumor immunosuppressive reprogramming (Figure 4H–K). These results suggest that *Nudt21* deficiency restricts CRC growth by augmenting CD8⁺ T cell antitumor immunity, at least in part through suppression of cholesterol biosynthesis.

### 2.5 Nudt21 Lengthens the 3′UTR of Cdk19 to Regulate Cholesterol Biosynthesis and CRC Proliferation

**Figure 5:**
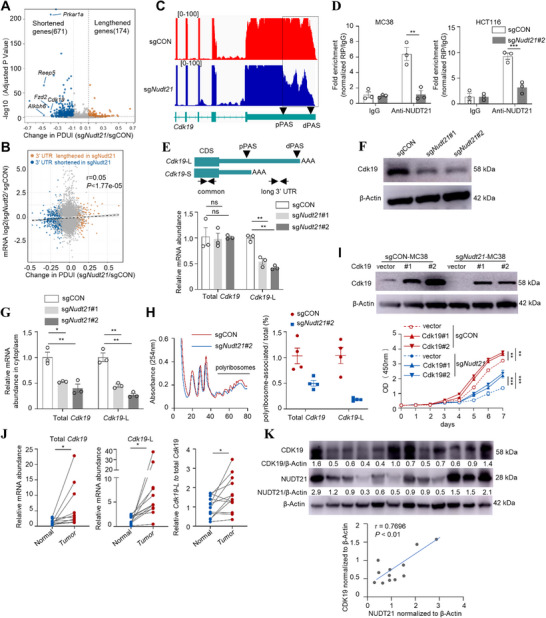
NUDTL21‐mediated selection of distant PAS of *Cdk19* to enhance its translation efficiency. A) 3′UTR APA changes in sgCON and sg*Nudt21* MC38 cells based on DaPars (|PDUI|>0.1, p < 0.05). Genes with significant 3′ UTR lengthening (red) or shortening (blue) are indicated. B) Correlation between dPAS usage and gene expression levels. The *x*‐axis shows the PDUI value. The *y*‐axis shows the logarithm of the gene expression levels from sg*Nudt21* MC38 cells relative to control cells. dPAS, distant PAS. C) RNA‐seq reads across the *Cdk19* loci in sgCON (red) and sg*Nudt21* (blue) MC38 cells. pPAS, proximal PAS. D) RIP‐qPCR demonstrating the binding of Nudt21 to *Cdk19* mRNA in MC38 or HCT116 cells expressing sgCON or sgNudt21 (n=3). E) Schematic illustrating the *Cdk19* long 3′ UTR isoform (*Cdk19*‐L) and short 3′ UTR isoform (Cdk19*‐S*). The primers targeting long 3′ UTR or common region (coding sequence, CDS) are indicated. RT‐qPCR analysis of relative mRNA expression of *Cdk19*‐L and total *Cdk19* in sgCON or sg*Nudt21* MC38 cells (n=3). F) Western blot analysis of Cdk19 protein expression in sgCON and sg*Nudt21* MC38 cells. G) RT‐qPCR analysis of *Cdk19*‐L and total *Cdk19* mRNA abundance in the cytoplasm of sgCON and sg*Nudt21* MC38 cells (n=3). H) Representative trace of ribosome extract prepared from sgCON and sg*Nudt21* MC38 cells in the presence of cycloheximide. RT‐qPCR analysis of ribosome occupancy of *Cdk19*‐L and total *Cdk19* mRNA in sgCON and sg*Nudt21* MC38 cells, measured as the relative expression ratio of polyribosome mRNAs to input mRNAs (n=4). I) Western blotting assay showing the protein levels of CDK19 in sgCON and sg*Nudt21* MC38 cells expressing ectopic Cdk19 (top). Cell growth curves of these cells were shown at the bottom (n=5). J) RT‐qPCR analysis of *Cdk19*‐L and total *Cdk19* mRNA abundance in 12 paired human CRC tumor specimens and their adjacent normal tissues. The ratio of *Cdk19*‐L to total *Cdk19* was calculated. K) Immunoblotting analysis of NUDT21 and CDK19 expression in human CRC tumor tissues. Correlation between NUDT21 and CDK19 expression levels was measured by Spearman's correlation analysis. All data are shown as mean ± SEM. Statistically significant level was indicated based on paired Student's *t* test (J), Welch's test (D and H), or one‐way ANOVA (E and G) and two‐way ANOVA (I). ^*^ *p* < 0.05, ^**^ *p* < 0.01, ^***^ *p* < 0.001, ns, no significance.

Nudt21 binds UGUA motifs proximal to dPASs to favor long 3′UTR isoforms production.^[^ ^29^, ^30^ ^]^ To delineate global APA alterations following Nudt21 loss in CRC, we applied the DaPars algorithm to transcriptomic data from *Nudt21*‐knockdown and control MC38 cells. DaPars can infer the de novo proximal PASs (pPASs) as well as the long and short 3′UTR expression levels, as indicated by the distal polyA site usage index (PDUI) value. We found that *Nudt21* knockdown led to 108 genes with 3′UTR lengthening and 464 with shortening (*P* < 0.05, ΔPDUI≥ 0.1) (**Figure** **5**A). Although 3′UTR shortening is expected to remove miRNA‐binding sites and increase stability and accumulation of the affected transcripts,^[^ ^31^ ^]^ global expression of the total *Cdk19* transcript was not significantly increased with its shortened 3′UTR isoforms in *Nudt21*‐knockdown MC38 cells (Figure 5B). Strikingly, no significant APA changes of cholesterol biosynthesis genes were detected in *Nudt21*‐deficient MC38 cells, such as *Srebf2, Hmgcs1, Cyp51*, and *Sqle* (Figure S6, Supporting Information), suggesting that Nudt21 controls their expression indirectly. Instead, we observed a pronounced loss of the long 3′UTR isoform of *Cdk19* (Figure 5C). *Cdk19*, along with its paralogue *Cdk8*, encodes the mediator‐associated kinase, which regulates gene transcription by RNA polymerase II (RNAPII).^[^ ^32^ ^]^ This observation raises the possibility that Cdk19 constitutes a pivotal mediator linking Nudt21‐driven APA regulation to transcriptional reprogramming in CRC. We next investigated whether Nudt21 directly controls *Cdk19* mRNA fate. RNA immunoprecipitation (RIP)‐QPCR assay confirmed a direct interaction between Nudt21 protein and *Cdk19* mRNA in *Nudt21*‐sufficient HCT116 and MC38 cells, which was markedly reduced upon *Nudt21* depletion (Figure 5D). To investigate dPAS usage in the *Cdk19* transcript, we designed two primer pairs targeting either the long 3′UTR or the coding sequence (CDS) of *Cdk19* mRNA (Figure 5E). The distal primer was designed to target sequences just before the dPASs and detect Cdk19 long 3′UTR transcripts (*Cdk19‐*L). The common primer was designed to target the CDS and was used for detecting the total transcript level (total *Cdk19*), including both *Cdk19*‐L and Cdk19 short 3′ UTR isoform (*Cdk19*‐S). Despite *Cdk19*‐L was selectively reduced in *Nudt21*‐deficient MC38 cells, total *Cdk19* transcript levels remained unchanged, yet its protein abundance was markedly diminished (Figure 5F), suggesting that 3′UTR lengthening enhances translation of Cdk19. Long 3′UTRs are known to regulate mRNA export from the nucleus to the cytoplasm and recruitment to polyribosomes.^[^ ^33^ ^]^ We next examine whether the observed mismatch between Cdk19 transcript abundance and protein expression results from altered cytoplasmic localization and reduced translation efficiency. Indeed, *Nudt21* depletion reduced the cytoplasmic abundance of both *Cdk19*‐L and total *Cdk19* mRNAs (Figure 5G), accompanied by decreased binding of total *Cdk19* transcripts to polyribosomes (Figure 5H). These results suggest that NUDT21 promotes *Cdk19* long 3′UTRs generation, thereby facilitating mRNA export and efficient protein synthesis.

We next investigated whether reduced *Cdk19* contributes to the proliferative defects in *Nudt21*‐deficient CRC cells. Treatment with a selective Cdk19 inhibitor in CRC cell lines and patient‐derived organoids (PDOs) significantly impaired proliferation (Figure S7A,B, Supporting Information) and downregulated cholesterol biosynthesis‐related gene expression (Figure S7C, Supporting Information), highlighting a role of Cdk19 for promoting tumor growth through metabolic reprogramming. Conversely, ectopic Cdk19 expression enhanced MC38 proliferation and substantially rescued the growth defect caused by *Nudt21* depletion (Figure 5I). To determine whether Cdk19 directly regulates the transcription of cholesterol synthesis genes, we performed CUT&RUN followed by qPCR in MC38 cells expressing Flag‐tagged CDK19. CDK19 was enriched at the promoter regions of key cholesterol synthesis genes, including *Srebf2, Hmgcs1*, and *Sqle* (Figure S7D, Supporting Information). Notably, phospho‐RNAPII (Ser5) occupancy at these loci was significantly reduced in *Nudt21*‐deficient MC38 cells compared to control cells (Figure S7E, Supporting Information). Collectively, these results identify CDK19 as a key downstream effector of NUDT21 that reprograms cholesterol metabolism by directly modulating RNAPII activity on cholesterol synthesis genes, likely through promoting transcriptional elongation.

To determine the association between the NUDT21‐CDK19 axis and cholesterol biosynthesis as well as antitumor responses in CRC, we analyzed TCGA datasets. We observed a positive correlation between NUDT21 and CDK19 expression levels in CRC tissues (Figure S8A, Supporting Information). Like NUDT21 (Figure 3F), we also found the positive correlation between *CDK19* expression and the cholesterol synthetic gene signature in TCGA datasets (Figure S8B, Supporting Information). Although neither NUDT21 nor CDK19 levels were associated with CD8^+^ T cell infiltration (Figure S8C, Supporting Information), both genes predicted T cell exhaustion signatures (Figure S8D, Supporting Information). Kaplan‐Meier survival analysis demonstrated that high CDK19 expression was associated with worse overall survival in CRC patients (Figure S8E, Supporting Information). In summary, these findings provide evidence for the importance of the NUDT21‐CDK19 axis in cholesterol biosynthesis, T cell exhaustion, and antitumor immunity in CRC.

Consistent with prior reports of CDK19 upregulation in CRC,^[^ ^34^ ^]^ we observed elevated total *Cdk19* and, more prominently, *Cdk19*‐L transcripts in tumors versus matched normal tissues (Figure 5J), suggesting that APA manner contributes to its aberrant regulation. Immunoblotting of human CRC samples further revealed a positive correlation between NUDT21 and CDK19 protein abundance (Figure 5K). In summary, these findings indicate that NUDT21 promotes *CDK19* expression through APA‐mediated upregulation of *CDK19*‐L, positioning CDK19 as a key effector of NUDT21.

### 2.6 Truncation of 3′ UTR Cdk19 Isoform Suppresses CRC Proliferation and Cholesterol Biosynthesis

**Figure 6:**
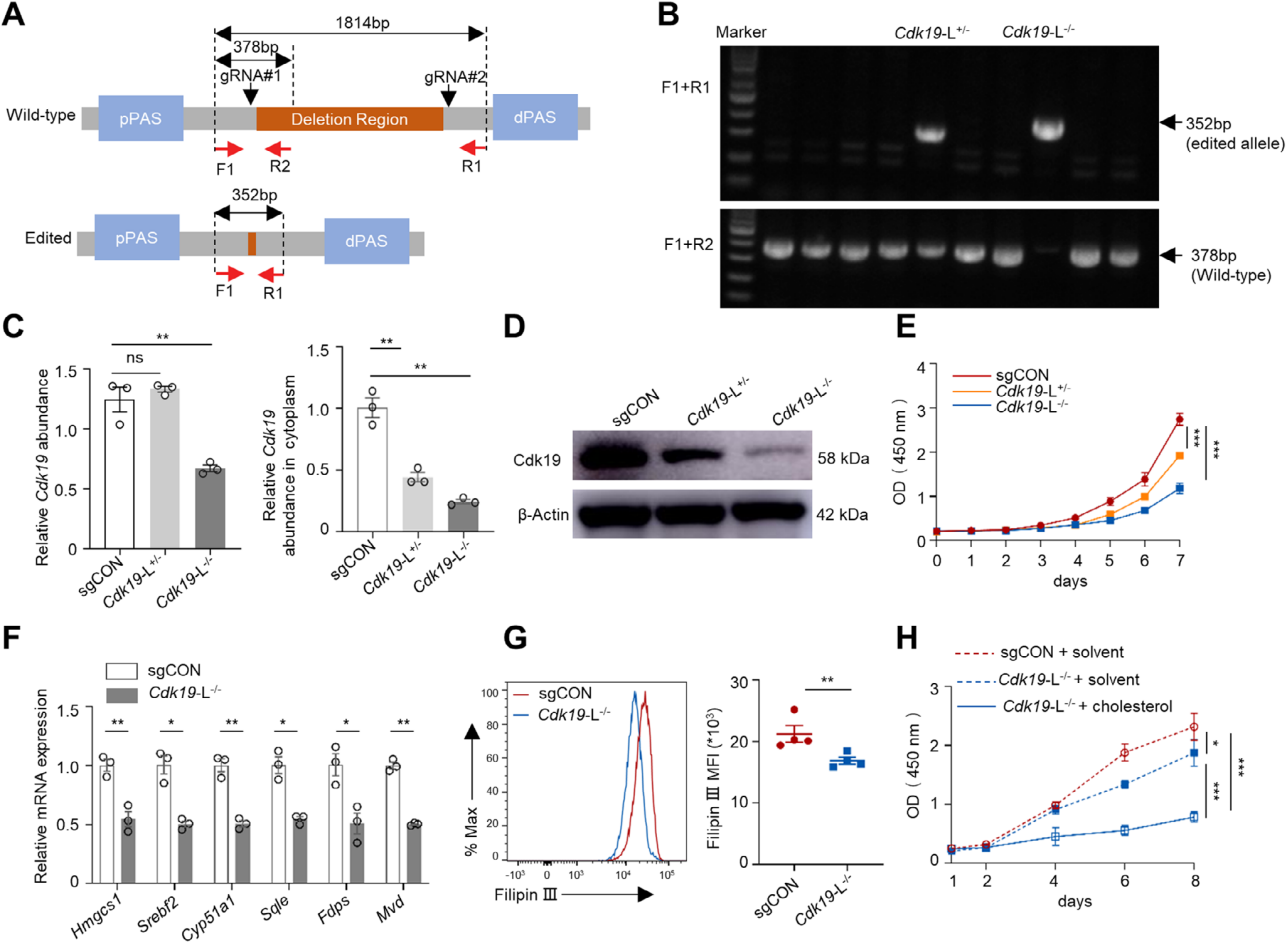
Truncation of the long 3′UTR of *Cdk19* suppresses CRC proliferation and cholesterol biosynthesis. A) A schematic of CRISPR/Cas9‐mediated deletion of the long 3′UTR of *Cdk19*. Two sgRNAs targeting the region downstream of pPAS and upstream of dPAS were used to remove the long 3′UTR (brown). Primers used for clone screening are shown in red, with the expected PCR product size indicated. B) PCR genotyping of MC38 clones following CRISPR/Cas9‐mediated targeting. One monoallelic (*Cdk19*‐L^⁺/−^) and one biallelic (*Cdk19*‐L^−/−^) deletion of the long 3′UTR of the *Cdk19* clone were identified. C) RT‐qPCR analysis of total *Cdk19* abundance in whole cell lysis (left) and cytoplasm (right) in indicated MC38 clones (n=3). D) Western blot analysis of Cdk19 protein levels in the indicated MC38 clones. E) CCK‐8 analysis of sgCON, *Cdk19*‐L^⁺/−^ and *Cdk19*‐L^−/−^ MC38 cells (n=5). F) RT‐qPCR analysis of cholesterol biosynthesis‐related gene expression in sgCON or *Cdk19*‐L^−/−^ MC38 cells (n=3). G) FACS analysis of Filipin III staining of cholesterol in sgCON or *Cdk19*‐L^−/−^ MC38 cells (n=4). (H) CCK‐8 analysis of sgCON or *Cdk19*‐L^−/−^ MC38 cells cultured with or without exogenous cholesterol (n=5). All data are shown as mean ± SEM. Statistically significant level was indicated based on Welch's test (F and G) or one‐way ANOVA (C) and two‐way ANOVA (E and H). ^*^ *p* < 0.05, ^**^ *p* < 0.01, ^***^ *p* < 0.001.

To probe the functional contribution of the long 3′UTR in regulating Cdk19 expression, we used the CRISPR/Cas9 system to delete the genomic region spanning the proximal and distal PASs in MC38 cells (**Figure** **6**A). Genomic PCR identified clones with monoallelic or biallelic deletions (designated *Cdk19*‐L^⁺/−^ and *Cdk19*‐L^−/−^, respectively) (Figure 6B). *Cdk19*‐L^−/−^ clones exhibited a ≈50% reduction in total *Cdk19* mRNA levels, whereas *Cdk19*‐L^⁺/−^ clones maintained total transcript abundance (Figure 6C, left). In both cases, cytoplasmic *Cdk19* mRNA was markedly reduced (Figure 6C, right), leading to diminished protein expression (Figure 6D). These results demonstrate that the long 3′UTR of *Cdk19* is required for efficient mRNA export and translation.

Both *Cdk19*‐L^⁺/−^ and *Cdk19*‐L^−/−^ clones exhibited impaired proliferation, with a more pronounced defect in *Cdk19*‐L^−/−^ cells (Figure 6E), indicating a dose‐dependent requirement of Cdk19 for cell growth. *Cdk19*‐L^−/−^ clones also showed downregulation of cholesterol biosynthesis genes (Figure 6F) and lower cholesterol content (Figure 6G). Notably, exogenous cholesterol supplementation rescued the proliferation defect of *Cdk19*‐L^−/−^ clones (Figure 6H). Together, these findings demonstrate that the long 3′UTR is required to maintain Cdk19 expression, which in turn drives cholesterol biosynthesis.

### 2.7 Deletion of 3′UTR Cdk19 Isoform Reactivates Antitumor CD8^+^ T Cell Immunity

**Figure 7:**
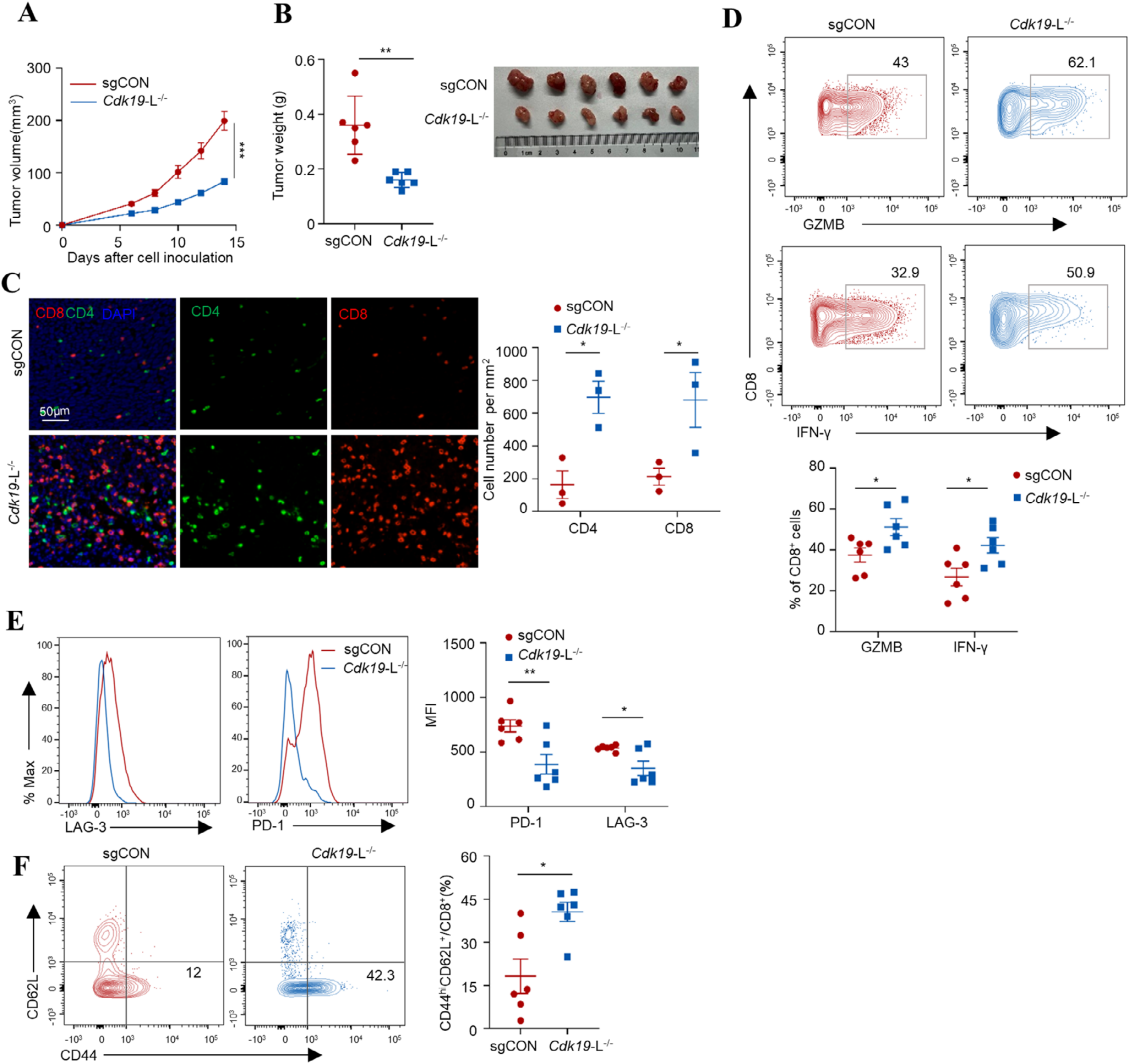
Loss of 3′UTR *Cdk19* isoform reactivates anti‐tumor CD8^+^ T cell activity. A, B) Growth curve and tumor weight measurement of subcutaneous sgCON or *Cdk19*‐L^−/−^ MC38 tumors in C57BL/6J mice. C) Representative mIHC images and quantification of CD4^+^ and CD8^+^ T cells from subcutaneous sgCON or *Cdk19*‐L^−/−^ MC38 tumors (n=3). Scale bar: 50 µm. D) Representative flow plots and quantification showing the proportion of GZMB‐ and IFN‐γ‐producing CD8^+^ T cells from subcutaneous sgCON or *Cdk19*‐L^−/−^ MC38 tumors (n=6). E) Representative flow plots and quantifications showing the surface PD‐1 and LAG‐3 expression in CD8^+^ T cells from subcutaneous sgCON or *Cdk19*‐L^−/−^ MC38 tumors (n=6). F) Representative flow plots and quantifications showing the proportions of effector memory/effector (CD44^hi^CD62L^−^) subset in CD8^+^ T cells from subcutaneous tumors (n=6). Each symbol represents an individual mouse. All data are shown as mean ± SEM. ^*^ *p* < 0.05, ^**^ *p* < 0.01 by Welch's test (B, C, D, E, and F) or two‐way ANOVA (A).

We next examined whether loss of the long 3′UTR of *Cdk19* recapitulates the tumor‐suppressive effects of *Nudt21* deficiency in syngeneic tumor models. Ablation of the long 3′UTR of *Cdk19* in MC38 cells resulted in impaired tumor growth (**Figure** **7**A,B), and enhanced tumor‐infiltrating CD8⁺ and CD4⁺ T cells (Figure 7C). CD8⁺ T cells from *Cdk19*‐L^−^/− tumors had higher frequencies of GZMB^+^ and IFN‐γ^+^ proportions (Figure 7D), compared with control counterparts. Moreover, they exhibited lower PD‐1 and LAG‐3 expression (Figure 7E). In addition, *Cdk19*‐L^−/−^ tumors exhibited an increased frequency of effector memory/effector CD8⁺ T cells (CD44^hi^CD62L^−^) (Figure 7F). These findings suggest that reduced *Cdk19* expression resulting from long 3′UTR truncation enhances anti‐tumor CD8⁺ T cell responses, mirroring the immune phenotype observed in *Nudt2*1‐deficient tumors.

## 3 Discussion

Dysregulated APA is emerging as a hallmark of cancer, yet its functional relevance in CRC progression has remained elusive. In this study, we systematically identified key APA regulators critical for CRC progression through a CRISPR/Cas9‐based functional screening. Among these candidates, NUDT21 emerged as a top candidate whose depletion markedly suppressing CRC cell growth in vitro. Mechanistically, NUDT21 drives cholesterol biosynthesis, thereby fueling tumor cell proliferation and attenuating anti‐tumor immunity. Moreover, these effects depended on NUDT21‐mediated lengthening of *Cdk19* 3′UTR and enhanced CDK19 translation. Together, our findings identify NUDT21 as a critical driver of CRC pathogenesis and uncover an unrecognized link between APA dysregulation, cholesterol metabolic reprogramming, and immune evasion, thereby providing new insight into the multifaceted role of post‐transcriptional on tumor biology regulation.

NUDT21 has been reported to exert conflicting roles across different human malignancies, functioning as either a tumor promoter or suppressor depending on cellular context.^[^ ^35^ ^]^ NUDT21 was overexpressed in clinical CRC tumor tissues compared to adjacent normal mucosa, and its higher expression predicts poor prognosis. NUDT21 promotes CRC progression by simultaneously accelerating tumor growth and dampening anti‐tumor immunity. Previous studies have highlighted the importance of APA in shaping cancer transcriptomes, often focusing on oncogene activation or tumor suppressor expression through 3′UTR mechanisms,^[^ ^36^ ^]^ supporting the context‐dependent role of NUDT21 across various cancers, presumably by acting on different targets.

Our study uncovers NUDT21 as an essential regulator of cholesterol metabolism that underpins CRC progression. Nudt21 deletion markedly reduced the expression of key cholesterol biosynthesis genes (*Hmgcs1, Sqle, Cyp51a1, Fdps, Mvd, and Srebf2*) and lowered intracellular cholesterol content in CRC cells. It was well‐known that the rapidly malignant tumor cells required abundant cholesterol for membrane synthesis and cellular signaling.^[^ ^37^ ^]^ Cholesterol supplementation restored the proliferation of *Nudt21*‐deficient cells, indicating that NUDT21 promotes tumor growth in part by enhancing cholesterol biosynthesis. Metabolomic profiling extended these observations, showing that *Nudt21*‐deficient tumors had reduced cholesterol and its derivatives, including oxysterols and lanosterol. Emerging evidence has demonstrated that tumor‐derived cholesterol and its metabolites restrain anti‐tumor CD8^+^ T cell immune responses.^[^ ^28^ ^]^ Constantly, *Nudt21*‐deficient tumors displayed increased CD8⁺ T cell infiltration and effector functions, accompanied with alleviating exhaustion features. Pharmacological inhibition of cholesterol synthesis impaired tumor growth and augmented CD8⁺ T cell responses, with a more pronounced effect in *Nudt21*‐proficient tumors. We also showed that NUDT21 deficiency in CRC enhances CD8^+^ T cell activity, partially through cholesterol reduction. It is expected that the reduction of cholesterol derivatives may augment CD8⁺ T cell antitumor immunity upon NUDT21 inhibition. Together, these findings identify tumor‐intrinsic NUDT21 as a driver of cholesterol biosynthesis, linking metabolic rewiring to anti‐tumor CD8^+^ T immunity in CRC. This observation not only advances our understanding of CRC pathogenesis but also opens avenues for exploiting metabolic‐immune crosstalk in treatment strategies for CRC.

NUDT21 loss has been reported to cause global 3′UTR shortening and stabilize the affected transcripts.^[^ ^38^, ^39^ ^]^ In MC38 cells, however, Nudt21 knockdown did not induce widespread 3′UTR alterations, suggesting functional redundancy in APA regulation and a dose‐dependent effect. Notably, Nudt21 deletion reduced the expression of cholesterol biosynthesis genes without altering their 3′UTR length, but selectively shortened the 3′UTR of *Cdk19* and decreased Cdk19 protein abundance. Unlike canonical cyclin‐dependent kinases, Cdk19 acts as a transcriptional co‐activator within the mediator complex and is frequently dysregulated in cancers, where it promotes proliferation and therapy resistance.^[^ ^32^, ^40^ ^]^ We found that CDK19 binds directly to the promoter regions of cholesterol synthesis genes (Srebf2, Hmgcs1, and Sqle) and NUDT21 deficiency reduced the occupation of phosphorylated RNAPII on these loci, suggesting that the NUDT21‐CDK19 axis directly regulates their transcription. Given that SREBF2 is a master transcriptional regulator of cholesterol synthesis genes (*Hmgcs1* and *Sqle*),^[^ ^41^ ^]^ we propose that CDK19 also indirectly upregulates Hmgcs1 and Sqle expression via Srebf2. Cdk19 inhibition mirrored the transcriptional changes induced by *Nudt21* loss, whereas Cdk19 re‐expression restored cell proliferation and cholesterol biosynthesis‐related gene expression. Truncation of the long 3′UTR *Cdk19* isoforms phenocopied *Nudt21* loss, recapitulating the defects in cholesterol biosynthesis and the activation of anti‐tumor immunity. These findings indicate that NUDT21 does not directly elevate cholesterol biosynthesis gene expression but promotes the generation of long 3′UTR *Cdk19* isoforms.

3′UTRs are well‐known to regulate post‐transcriptional processes, including transcript stability, localization, nuclear‐cytoplasm export, and translation efficiency.^[^ ^42^ ^]^ We observed that Nudt21 deficiency mediated shortening 3′UTR of *Cdk19*, leaving total *Cdk19* mRNA levels unchanged but markedly reducing its cytoplasmic abundance and polyribosome engagement revealed by polyribosome profiling. Compared with the shorter isoforms, the long 3′UTR *Cdk19* transcript harbors a higher density of UGUA motifs, which are predicted to facilitate nuclear‐cytoplasm export.^[^ ^43^ ^]^ We predict that Nudt21‐mediated lengthening 3′UTR of *Cdk19* facilitates its mRNA cytoplasmic export and increases translational availability. We cannot exclude, however, that the long 3′UTR of *Cdk19* also contains elements that directly enhance polyribosome recruitment and translational efficiency.

Currently, a series of small‐molecule inhibitors targeting CDK19 is under development for hyperproliferative tumors.^[^ ^44^ ^]^ Our findings highlight an additional layer of therapeutic relevance by showing that CDK19 blockade delays tumor development, not only directly impairing tumor proliferation but also indirectly enhancing anti‐tumor T cell immunity by disrupting cholesterol biosynthesis. These findings suggest that combining CDK19 inhibitors or statins with immune checkpoint blockade could synergistically enhance anti‐tumor responses in patients with CRC. While inhibition of the NUDT21‐CDK19 axis shows promising anti‐tumor effects in preclinical CRC models, several limitations remain. First, the role of NUDT21 in immune cells was not explored in this study. Further work is needed to define its immune‐related functions before considering NUDT21 inhibitors in clinical use. Second, because complete NUDT21 ablation was lethal in MC38 cells, this study mainly relied on NUDT21‐knockdown models. Future studies should employ CRC cell lines with full NUDT21 deletion to comprehensively dissect its functions and mechanisms of action.

## 4 Conclusion

In conclusion, our research highlights NUDT21 as a pivotal regulator that directs dPAS usage of *Cdk19*, contributing to cell proliferation and immunosuppressive reprogramming via modulation of cholesterol metabolism. This work establishes an unrecognized mechanistic link between APA and cholesterol biosynthesis regulation, expanding the current knowledge of post‐transcriptional control in cancer and suggesting the NUDT21‐CDK19‐cholesterol axis as a promising therapeutic vulnerability in CRC.

## 5 Experimental Section

### Human Specimens

Surgically resected human CRC specimens, along with paired adjacent normal tissues, were collected from Huashan Hospital, Fudan University. None of the participants had received anti‐tumor therapies or undergone surgical treatment prior to specimen collection. The study protocol was approved by the Institutional Research Ethics Committee of Huashan Hospital (Ethical number KY2022‐1095), and written informed consent was obtained from all patients before they could participate.

### Cell Lines

The human CRC cell lines (HCT116 and HT29), murine CRC cell line MC‐38, and HEK‐293T cells were sourced from the American Type Culture Collection (ATCC). These cells were cultured in McCoy's 5A medium (Gibco) or Dulbecco's modified Eagle's medium (DMEM, Corning) with 10% fetal bovine serum (FBS, ExCell Bio) and 1% penicillin‐streptomycin solution (PS, NCM Biotech). The identity of these Cell lines was validated using short tandem repeat genotyping, and mycoplasma contamination was ruled out by using a Mycoplasma PCR detection method.

### Mice

Female C57BL/6J mice aged six to eight weeks were purchased from Shanghai GlinX Biotechnology Co., Ltd. All mice were housed under pathogen‐free conditions with a 12‐h light/12‐h dark cycle. All mouse experiments were conducted under authorization of the Institutional Animal Care and Use Committee (IACUC) of Shanghai Jiao Tong University School of Medicine.

### CRISPR/Cas9 Deletion Library Screening

To systematically interrogate RNA regulatory pathways in CRC, a customized CRISPR/Cas9 library targeting 98 RNA‐regulatory genes was designed (Table S1, Supporting Information) with 428 sgRNAs synthesized by Xunhong Co., Ltd. (Suzhou, China). Lentiviral particles were generated by transfecting HEK‐293T cells with the library plasmids and subsequently used to infect HCT116 cells at a low multiplicity of infection (MOI ≈0.3) for 72 h, ensuring uniform sgRNA representation. Selection under puromycin (1 µg mL^−1^, Beyotime) pressure ensued for 96 h, a step generating cell pools with stable sgRNAs expression. These pools were split, with one harvested as the baseline control and the other cultured for an additional 14 days. Genomic DNA from harvested cells was extracted using a DNA Extraction Kit (Tiangen) and subjected to amplify the sgRNA regions by PCR for next‐generation sequencing. MAGeCKFlute was used to process sgRNA read counts from sequencing data, yielding sgRNAs significantly enriched or depleted between conditions.^[^ ^45^ ^]^

### Generation of NUDT21 Knockout Cell Lines

For NUDT21 knockout cell lines, independent sgRNAs targeting NUDT21 were designed and cloned into vector pLenti‐CRISPRv2 (Addgene, #52961). sgRNAs sequences are listed in Table S2, Supporting Information. HCT116, HT29, and MC38 cells were transfected with sgNudt21 or sgCON lentivirus and selected with puromycin for 3 days. Single‐cell‐derived colonies verified for NUDT21 loss were expanded and used for subsequent experiments.

### Generation of CDK19 Long 3′UTR Knockout MC38 Cells Using CRISPR/Cas9 Technology

To specifically ablate the long 3′UTR isoform of *Cdk19* in MC38 cells, a CRISPR/Cas9 strategy was employed, designed to reposition the dPAS in close proximity to the pPAS, thereby favoring proximal cleavage and polyadenylation.^[^ ^46^ ^]^ Two sgRNAs were designed to target regions downstream of the pPAS and upstream of the dPAS (Figure 6A), with sequences provided in Table S2, Supporting Information. Each sgRNA was cloned into the pSpCas9(BB)‐2A‐GFP plasmid (Addgene, #48138), and co‐transfection was performed with pSpCas9(BB)‐2A‐Puro (Addgene, #48139) at a 10:10:1 ratio using Lipofectamine 3000 (Invitrogen). Following 3 days of puromycin selection, single‐cell‐derived colonies were isolated. Genomic DNA was extracted, and PCR genotyping was performed using primers listed in Table S2, Supporting Information, with the genotyping strategy illustrated in Figure 6A.

### Cell Proliferation and Colony Formation Assays

To evaluate the impact on tumor cell growth, proliferation, and clonogenic capacity were measured. For proliferation, 400 cells per well were seeded in 96‐well plates and cultured for 7 days, followed by viability assessment using CCK‐8 (YEASEN). For colony formation, 500 cells per well were seeded in 6‐well plates and maintained for 10 days. Colonies were fixed with 4% paraformaldehyde (Servicebio) and stained with 0.1% crystal violet at room temperature. Pharmacological inhibition was performed using a CDK19 inhibitor (SEL120‐34A hydrochloride, MedChemExpress) for 48 h, and the addition of exogenous cholesterol (75 µm, Selleck) was tested for its ability to attenuate growth suppression.

### Cell Cycle and Apoptosis Tests

Cell cycle distribution was assessed by 5‐Ethynyl‐2′‐deoxyuridine (EdU) incorporation and flow cytometry (EdU Cell Proliferation Assay Kit, RiboBio). Apoptosis was evaluated by Annexin V‐FITC/propidium iodide (PI) staining (FITC Annexin V Apoptosis Detection Kit I, BD Pharmingen). Cells and supernatants were collected, stained for 15 min at room temperature in the dark, and analyzed by flow cytometry within 1 h.

### Animal Tumor Models and In Vivo Treatments

For syngeneic CRC models, 5 × 10^5^ sgNudt21, long 3′UTR of Cdk19 knockout (Cdk19‐L^−/−^), or corresponding control MC38 cells were subcutaneously (s.c.) injected into the female C57BL/6J mice. Tumor size was measured every two days starting on day 6‐7 after inoculation, and tumor volume was calculated as 𝑉=(W^2^×H)/2. At day 14 after inoculation, mice were sacrificed for further analysis. Tumor maximum diameters remained below 15 mm throughout the study. Sample sizes are reported in figure legends. Kaplan‐Meier survival analysis was performed where indicated. For therapeutic evaluation, sgNudt21 or sgCON tumor‐bearing mice received intraperitoneal injections (i.p.) of simvastatin (25 mg kg^−1^, Selleck) every other day beginning on day 7 post‐inoculation.

### RNA Sequencing and APA Data Analysis

To investigate transcriptome‐wide expression and APA alternation, total RNA was extracted from sg*Nudt21* and sgCON MC38 cells using TRIzol Reagent (Invitrogen), followed by polyA‐enriched library preparation, and high‐depth sequencing (≈200 million reads per sample; Shanghai Neo‐Biotechnology).

The clean reads were aligned to the mouse reference genome (mm10, GRCm38) with HISAT2 (v2.2.1), and gene counts were derived using FeatureCounts (v1.28.1). DEGs were defined at *P* < 0.05 with thresholds of log2 fold‐change ≥ 2.5 (upregulated) or ≤ −1.5 (downregulated). Functional enrichment analyses were interrogated by GO and GSEA. To capture APA regulation, BAM files were processed with DaPars () to estimate the percentage of PDUI values, providing a quantitative measure of dPAS usage.^[^ ^47^ ^]^ Significant APA events were identified as *P* < 0.05 and |ΔPDUI| > 0.1.

### Cholesterol Metabolomics

Tumor interstitial fluid from sg*Nudt21* and sgCON MC38 tumor tissues was subjected to cholesterol metabolomics by LC‐MS/MS. Samples were processed through sterol extraction, hydrolysis, derivatization, and cleanup. Briefly, 200 µL of interstitial fluid was mixed with 800 µL of sterol extraction solvent (DCM: MeOH = 2:1, v/v) containing 6.5 mg BHT, and metabolites were analyzed on an Agilent 6560 DTIM‐QTOFMS coupled with an Agilent 1290 UHPLC system. To account for sample heterogeneity, 20 µL of each interstitial fluid was reserved for protein quantification, and metabolite abundances were normalized to protein levels using established internal standards.^[^ ^48^ ^]^

### Cholesterol Content Measurement

In vitro cultured cells or tumor‐derived single‐cell suspensions were stained with Filipin III (Absin) and analyzed by flow cytometry. Absolute cellular cholesterol content was quantified in parallel using the Amplex Red Cholesterol Assay Kit (Beyotime), performed according to the manufacturer's instructions.

### Flow Cytometry Analysis

Fresh subcutaneous tumors were minced, enzymatically digested in medium containing 2% FBS, 1 mg mL^−1^ collagenase IV (Sigma), and 0.1 mg mL^−1^ DNase I (Roche) at 37 °C for 40 min, and filtered through 70 µm strainers (Corning). Single‐cell suspensions were blocked with anti‐FcR (BD Bioscience) for 15 min and stained with antibodies against surface markers at 4 °C for 30 min. For cytokine analysis, cells were stimulated with Cell Stimulation Cocktail plus protein transport inhibitor (eBioscience) for 5 h in the incubator prior to fixation, permeabilization, and intracellular antibody staining. The following antibodies were used in this study: anti‐CD45 (BioLegend, #30‐F11), anti‐CD4 (BioLegend, #GK1.5), anti‐CD8a (BioLegend, #53‐6.7), anti‐Ly6C (BioLegend, #HK1.4), anti‐Ly6G (BioLegend, #1A8), anti‐CD11b (eBioscience, #M1/70), anti‐CD11c (BioLegend, #N418), anti‐MHC class II (I‐A/I/E, BioLegend, #M5/114.15.2), anti‐NK1.1 (BioLegend, #PK136), anti‐F4/80 (BioLegend, #BM8), anti‐CD19 (eBioscience, #1D3), anti‐CD44 (BioLegend, #IM7), anti‐CD62L (eBioscience, #MEL‐14), anti‐PD‐1 (BD Bioscience, #MIH5), anti‐LAG‐3 (BioLegend, #C9B7W), anti‐GZMB (BioLegend, #GB11), and anti‐IFN‐γ (BD Bioscience, #XMG1.2). Samples were analyzed on an LSR X20 flow cytometer (BD Bioscience), and data analysis was performed by FlowJo v10. Gating strategies for flow cytometry analysis of tumors used in this study were illustrated in Figure S5, Supporting Information.

### T Cell Isolation and Coculture

T cells were isolated from female C57BL/6J mice using the EasySep Mouse CD8^+^ T Cell Isolation Kit (STEMCELL Technologies). Cells were stimulated and cultured with plate‐bound anti‐CD3ε (5 µg mL^−1^, eBioscience) and soluble anti‐CD28 (1 µg mL^−1^, eBioscience) antibodies in the presence of recombinant murine IL‐2 (10 ng mL^−1^, Sigma–Aldrich). After 2 days of differentiation, cells were cultured with or without the indicated treatments for another 48 h. Tumor supernatant (1 mg tumor in 1 mL T cell culture medium, filtered) was from s.c. grown sgNudt21 or sgCON MC38 tumors. CD8^+^ T cells were cultured with control T cell culture medium or with 100 µL tumor supernatant without or with Methyl‐β‐cyclodextrin (MβCD, 0.25 mm; Aladdin) as indicated.

### Multiplex Immunohistochemical (mIHC) Staining

Tumor sections were prepared from paraffin‐embedding and stained using a three‐color multiplex fluorescent IHC kit (Afantibody). Sections were deparaffinized, rehydrated, and subjected to antigen retrieval, then blocked and incubated overnight with primary antibodies. After washing, slides were incubated with HRP‐conjugated secondary antibodies and reagents for fluorescent signal amplification. For multiplex staining, antigen retrieval was repeated to remove bound antibodies before incubation with the next primary antibody. This process was repeated for each antigen. Sequential antigen retrieval and re‐staining steps enabled iterative labeling of different targets. Nuclei were counterstained with DAPI, and slides were mounted in an antifade reagent. High‐resolution images were captured with an Olympus SLIDEVIEW VS200 system.

### Quantitative Real‑Time PCR (RT‐qPCR)

Total RNA was extracted with TRIzol reagent and analyzed by RT‐qPCR using SYBR Green Master Mix (Vazyme). Relative expression was determined by the 2^–∆∆Ct^ method with β‐actin as the internal reference, providing normalization across samples. Primer sequences are listed in Table S2, Supporting Information. All assays were performed in biological duplicates to ensure robustness.

### RIP Assay

RIP assay was performed to assess RNA‐NUDT21 protein interactions as previously described.^[^ ^49^ ^]^ sg*Nudt21* or sgCON MC38 cells were lysed in a mild buffer (150 mm NaCl, 10 mm Tris, 0.1%NP‐40, pH 7.4) supplemented with RNase inhibitor (Roche). Antibodies against NUDT21 (1:100, Proteintech) or control IgG (1:100, Cell Signaling Technology) were pre‐bound to ProteinA/G Magnetic Beads (Thermo Scientific) for 3 h at 4 °C, then incubated with cell lysates overnight at 4 °C to capture RNA‐protein complexes. Then, the RNA‐protein IP complexes were washed and treated with proteinase K digestion buffer to remove the proteins. The coprecipitated RNA was purified using the RNeasy Micro Kit (QIAGEN) for RT‐qPCR analysis.

### Immunoblotting

Proteins were extracted using RIPA buffer (Beyotime) supplemented with protease and phosphatase inhibitors (Selleck). Protein concentrations were measured with the Pierce Assay Kit (Thermo Scientific). Immunoblotting was performed with primary antibodies against NUDT21, CDK19 (Sigma), and β‐actin (Beyotime).

### Polyribosome Profiling

Polyribosome profiling was performed following Esposito et al.^[^ ^50^ ^]^ sg*Nudt21* and sgCON MC38 cells (1 × 10^9^) were washed three times in cycloheximide (100 µg mL^−1^, MedChemExpress) on ice, and lysed in polysome lysis buffer (100 mm KCl, 0.1% Triton X‐100, 50 mm HEPES, 2 mm MgCl_2_, 10% glycerol, 1 mm dithiothreitol, 20 U mL^−1^ RNase inhibition, and 1× protease inhibitor cocktail). Lysates were clarified by centrifugation at 15,000 rpm at 4 °C for 15 min, and 20 µL was reserved as input RNA. The remainder was fractionated on 5‐50% linear sucrose gradients (10 mm Tris‐HCl, 5 mm MgCl_2_, 100 mm NaCl, and 1 mm dithiothreitol) by ultracentrifugation (SW60Ti rotor, Beckman) at 2 744 00 g for 2 h at 4 °C, with fractions collected using a Gradient Fractionator (Biocomp). RNA purified from input and polysome fractions (RNA Clean & Concentrator‐5, Zymo Research) was subjected to RT‐qPCR.

Total Cdk19 transcript levels (total *Cdk19*) were measured using CDS‐targeting primers, while Cdk19 long 3′ UTR isoforms (*Cdk19*‐L) were quantified with primers targeting the upstream of the dPAS (Figure 5E), enabling isoform‐specific assessment of *Cdk19* translational levels. Primer sequences are provided in Table S2, Supporting Information.

### Cytoplasmic RNA Preparation

For cytoplasmic RNA isolation, 2 × 10^6^ tumor cells were washed with cold PBS and harvested on ice. One‐fifth of the cells were reserved for whole‐cell RNA extraction, while the remainder was lysed in hypotonic buffer (10 mm HEPES, 1.5 mm MgCl_2_, 10 mm KCl, 0.2 mm PMSF, 0.5 mm dithiothreitol, and 0.075% NP‐40) on ice for 10 min to selectively permeabilize the plasma membrane. Following centrifugation at 2000 rpm for 5 min at 4 °C, the supernatant and pellet were collected as cytoplasmic and nuclear fractions, respectively. RNA from each fraction was extracted using TRIzol and analyzed by RT‐qPCR to assess subcellular transcript localization.

### PDO Culture and Drug Sensitivity Assay

Human CRC tissues were freshly resected and dissociated using a cocktail of collagenase IV (1 mg mL^−1^), DNase I (0.1 mg/mL), and hyaluronidase (0.1 mg mL^−1^, Sigma) at 37 °C for 20 min to obtain single cell suspension with viability. Cells were filtered through 70 µm strainers, centrifuged, and resuspended before embedding in Matrigel (Corning) in 24‐well plates. Organoids were cultured in a defined advanced DMEM/F12 (Gibco) medium containing HEPES, Glutamax (Gibco), FBS (Gibco), primocin (Gibco), noggin (R&D), hEGF (Invitrogen), Wnt‐3a (R&D), hR‐spondin‐1 (R&D), N‐2 MAX Media Supplement (R&D), B27 supplement (Gibco), nicotinamide (Sigma), Y‐27632 (YEASEN), N‐acetylcysteine (Sigma), A83‐01 (Sigma), SB202190 (Sigma), Gastrin I (MedChemExpress), and PEGF (Invitrogen).

### Overexpression of CDK19

To generate gene overexpression plasmids, the CDS sequence of Cdk19 was subcloned into pCDH‐EF1‐MCS‐T2A‐copGFP (CD521A‐1) vector. To establish stable cell lines, DNA constructs and lentivirus packaging plasmids were transfected into HEK‐293T cells. Lentivirus supernatants were used to infect sgCON or Sg*Nudt21*#2 MC38 cells. Two single‐cell‐derived colonies verified for CDK19 overexpression were expanded and used for subsequent experiments.

### CUT&RUN‐QPCR Assay

For CUT&RUN‐qPCR analysis, the CDS sequence of Cdk19 was cloned into the flag‐tagged pCDH‐EF1‐MCS‐T2A‐Puro (CD520A‐1) vector. MC38 cells were transfected with lentivirus supernatants produced from HEK‐293T cells. The transfected cells were subjected to selection with puromycin for 72 h and expanded for the CUT&RUN‐qPCR assay. The CUT&RUN‐qPCR assay was conducted using the Hyperactive pA/G‐MNase CUT&RUN Assay Kit for PCR/qPCR (HD103, Vazyme) following the manufacturer's protocol. Briefly, 5 × 10^5^ cells were harvested and adhered to Concanavalin A‐coated Magnetic Beads. sgCON and sg*Nudt21*#2 MC38 cells were treated with primary antibodies against phospho‐RNAPII (Ser5) (Abcam, #4H8, 1:100), while MC38 cells expressing Flag‐tagged Cdk19 or controls were treated with anti‐Flag (Sigma–Aldrich, #M2, 1:100) after being resuspended in antibody buffer. Then, the specimens were treated with pA/G‐MNase. Following segmentation, DNA was extracted and subjected to RT‐QPCR analysis. Primers targeting the promoter region of *Srebf2*, *Sqle*, *Hmgcs1*, and the non‐target gene were listed in Table S2, Supporting Information.

### Public Database Analysis

Transcriptomic data, methylation data, and copy‐number variation data for CRC were obtained from the Cancer Genome Atlas (TCGA)‐colon adenocarcinoma (COAD) dataset and TCGA‐rectal cancer (READ) dataset. Survival analysis was completed with the Kaplan‐Meier method. Proteomic data were obtained from the Clinical Proteomic Tumor Analysis Consortium (CPTAC) dataset (https://pdc.cancer.gov/pdc).

### Statistical Analysis and Reproducibility

All statistical analyses were performed using GraphPad Prism 10. Unless otherwise specified, experimental data were presented as the mean ± SEM from at least three independent biological replicates. For comparison between two groups, paired Student's t tests or Welch's t tests were performed. For comparison between more than two groups, one‐way or two‐way ANOVA was applied. For Kaplan‐Meier survival curves, the P values were calculated using the log‐rank test. The correlation was analyzed using a Pearson correlation test. Statistical significance was indicated as follows: ^*^ *P* < 0.05, ^**^ *P* < 0.01, ^***^ *P* < 0.001, and ns, no significance. The work model illustration was created using BioRender (https://www.biorender.com).

## Conflict of Interest

The authors declare no conflict of interest.

## Author Contributions

Y.Y., J.M., and M.Z. contributed equally to this work. Y.Y. conducted the in vivo and in vitro experiments, interpreted the data, and generated the figures. J.M. and M.Z. constructed plasmids and analyzed sequencing data. Y.Z. and W.G. assisted in the public datasets analysis. X.G. and T.Z. recruited participants and collected samples. Z.L. helped with the mouse colorectal tumor model. The original draft of the manuscript was written by Y.Y. and C.H., while Y.Y., W.G., and C.H. contributed to review and editing. J.X., C.H., and W.G. conceived the project and guided the experimental design. Supervision was provided by J.X., C.H., and W.G. All authors read and approve the final manuscript.

## Supporting information



Supporting Information

Supplemental Table 1

Supplemental Table 2

## Data Availability

The data that support the findings of this study are available from the corresponding author upon reasonable request.
