# Supplementary material for: NUDT21‐mediated Alternative Polyadenylation of CDK19 Reprograms Cholesterol Biosynthesis to Drive Colorectal Cancer Progression
Source: Adv Sci (Weinh). 2025 Nov 19;13(7):e18346. doi: 10.1002/advs.202518346 (PMC12866688; doi:10.1002/advs.202518346)
Supplement: Supplementary file 1 — Supporting Information [file ADVS-13-e18346-s002.pdf]

Supporting Information

NUDT21-mediated alternative polyadenylation of CDK19 reprograms cholesterol biosynthesis to drive colorectal cancer progression

Yeping Yu, Jixin Ma, Minwei Zhou, Xiaodong Gu, Yiming Zhou, Zhenyang Li, Tianyu Zhang, Wei Gong,* Chuanxin Huang,* and Jianbin Xiang *

	Num.	gene_name	Log2Fold- change	adj_P value	FDR
Negative correlation	1	NUDT21	-1.7524	0.000035	0.003391
	2	UPF1	-1.8106	0.00039459	0.019138
	3	CLP1	-1.1299	0.011173	0.317926
	4	PELO	-0.98219	0.013571	0.317926
	5	SMG5	-0.89285	0.016388	0.317926
	6	CSTF3	-0.88024	0.020124	0.325339
	7	FIP1L1	-0.99188	0.046607	0.593932
	8	SMG1	-0.905	0.048984	0.593932
Positive correlation	1	MSI2	0.66655	0.00053444	0.051841
	2	PABPC4L	0.45105	0.0037111	0.109133
	3	PABPC3	0.4498	0.0042706	0.109133
	4	UPF3B	0.48534	0.0045003	0.109133
	5	PPP2R2D	0.44432	0.0064982	0.126066
	6	PABPC1L2B	0.37086	0.01466	0.236999
	7	PYM1	0.31467	0.032641	0.452312
	8	PAPOLB	0.59933	0.044029	0.502315
	9	CSTF2T	0.48536	0.046607	0.502315

Figure S1. **MAGeCK analysis of significantly depleted or enriched genes using CRISPR-Cas9 screening.**

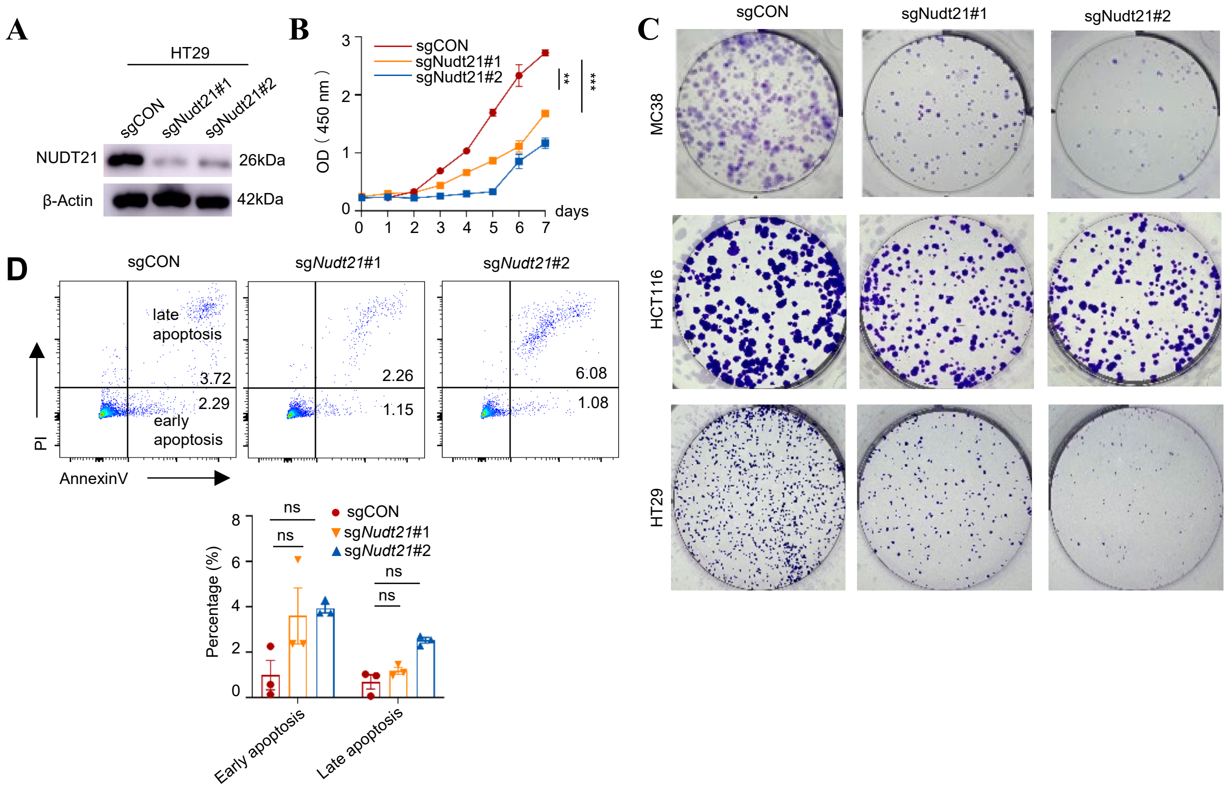

Figure S2. **NUDT21 ablation inhibits CRC proliferation.**

(**A**) Western blot showing the efficacy of sgRNA targeting Nudt21 in HT29 cells.

(**B**) Cell growth curve of sgCON or sgNudt21-HT29 cells (n=5).

(**C**) Colony formation of HCT116, MC38, and HT29 cells expressing sgCON or sgNudt21 (n=3).

(**D**) FACS analysis of Annexin V/propidium iodide (PI) staining in sgCON or sg*Nudt21*-MC38 cells. Percentage of early (Annexin V^{+} PI^{-}) or late (Annexin V^{+} PI^{+}) apoptosis cells was calculated (n=3).

Data represent the mean ± SEM. *p < 0.05, **p < 0.01; ns, not significant by one way ANOVA (**D**) and two-way ANOVA (**B**).

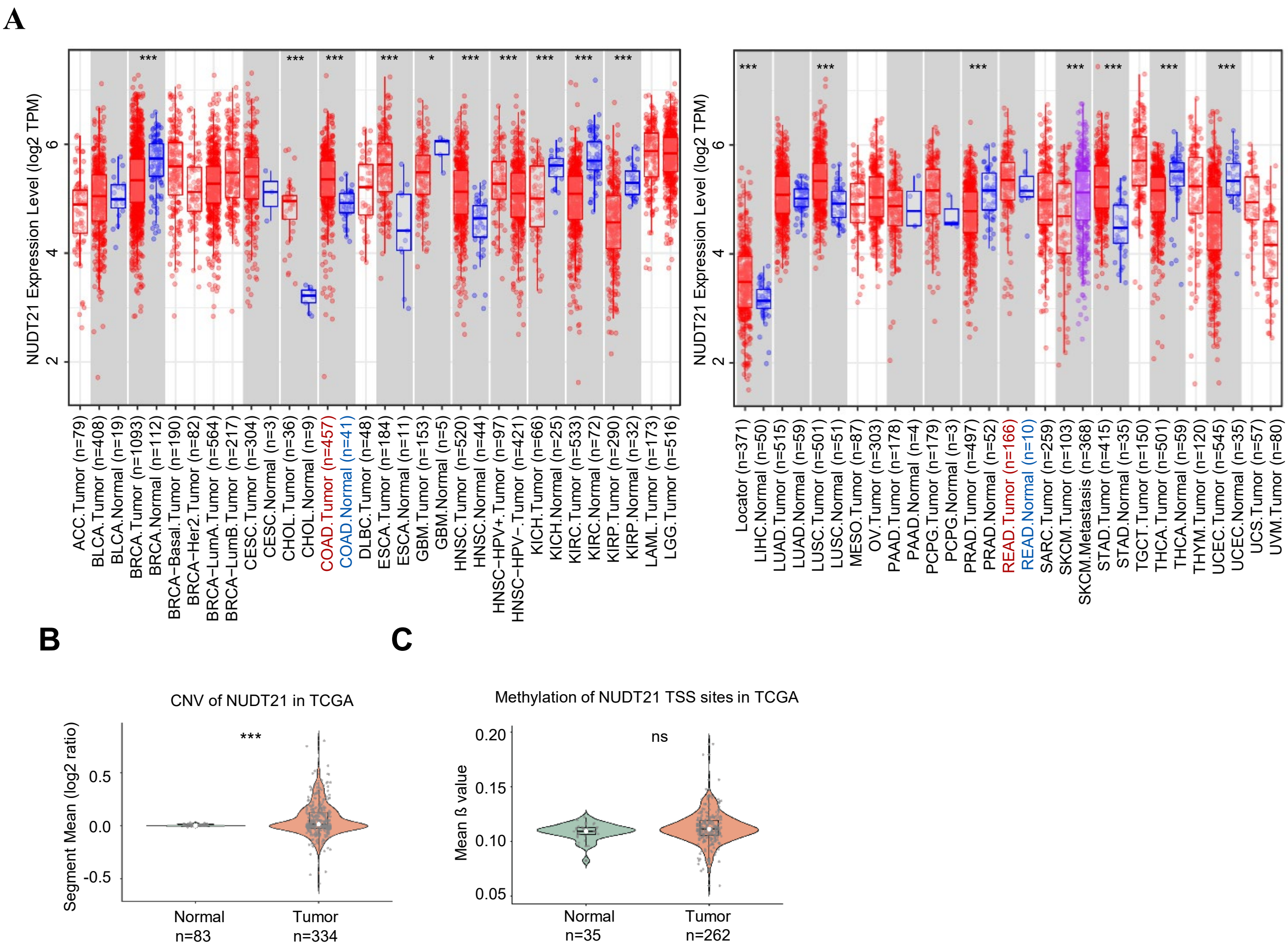

Figure S3. **NUDT21 expression pattern across human multiple cancers and genomic alterations in CRC.**

(**A**) Boxplots showing the relative NUDT21 mRNA levels in human tumor and matched normal tissues.

(B) Violin plot showing the CNV levels of *NUDT21* between CRC (n=334) and normal tissues (n=83) based on TCGA dataset

(**C**) Violin plot showing the methylation level of *NUDT21* in identified TSS sites between CRC (n=262) and normal tissues (n=35) based on TCGA dataset.

p values are determined by Welch's test **(B)** and Mann-Whitney U Test **(C)**. *p<0.05; **p<0.01; ***p<0.001; ns, not significant. CNV, copy number variation; TSS, transcription start site.

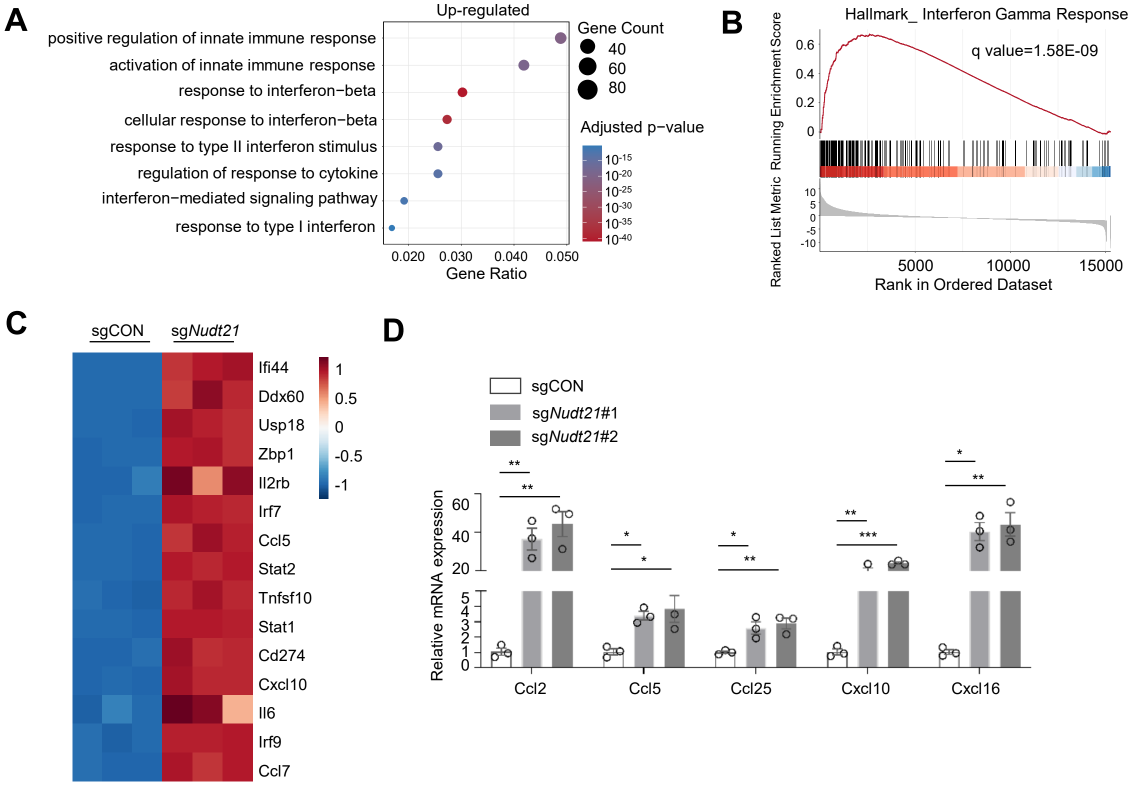

Figure S4. **NUDT21 deficiency activates IFN-γ response in MC38 cells.**

(**A**) Go enrichment pathway analysis of upregulated genes in sg*Nudt21*-MC38 cells compared to control cells.

(**B**) GSEA analysis of IFN-γ response signature in sgCON and sg*Nudt21* MC38 cells.

(**C**) Heatmap of IFN-γ response-related genes in sgCON and sg*Nudt21* MC38 cells.

(**D**) RT-qPCR analysis of selected IFN-γ response-related genes in sgCON and sg*Nudt21*-MC38 cells.

Data represents the mean ± SEM. *$p < 0.05$, **$p < 0.01$, ***$p < 0.001$ by one-way ANOVA **(D)**.

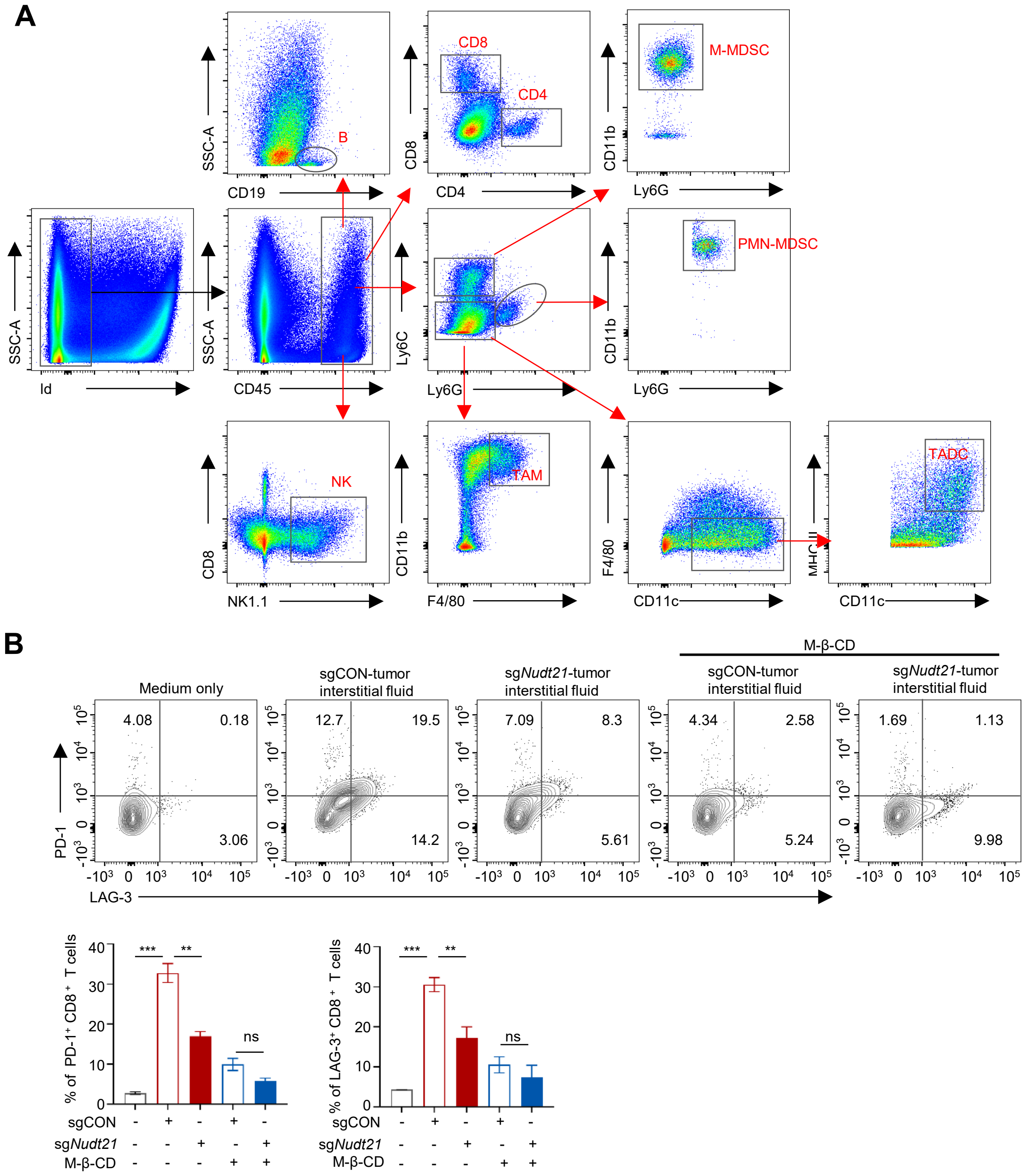

Figure S5. **Tumor interstitial fluid up-regulates LAG-3 and PD-1 expression on CD8⁺ T cells in vitro**

(A) Representative flow cytometry plots of tumor-infiltrating CD4⁺ T cells, CD8⁺ T cells, B cells, NK cells, TAMs, PMN-MDSCs, Mo-MDSCs, and TADCs for the indicated groups. TAMs, tumor associated macrophages; PMN-MDSC, polymorphonuclear myeloid derived suppressor cells; M-MDSCs, monocytic myeloid-derived suppressor cells; TADCs, tumor-associated dendritic cells.

(B) CD8⁺ T cells were in vitro stimulated with CD3/CD28 antibodies in the presence of IL-2 for 48 h, and cultured with indicated tumor interstitial fluid without or with Methyl-β-cyclodextrin (M-β-CD) for another 48 h for analysis. Representative flow cytometry plots of the expression of LAG-3 and PD-1 on CD8⁺ T cells and quantifications. Data represents the mean ± SEM. *p < 0.05; **p < 0.01; ***p < 0.001; ns, not significant by two-way ANOVA

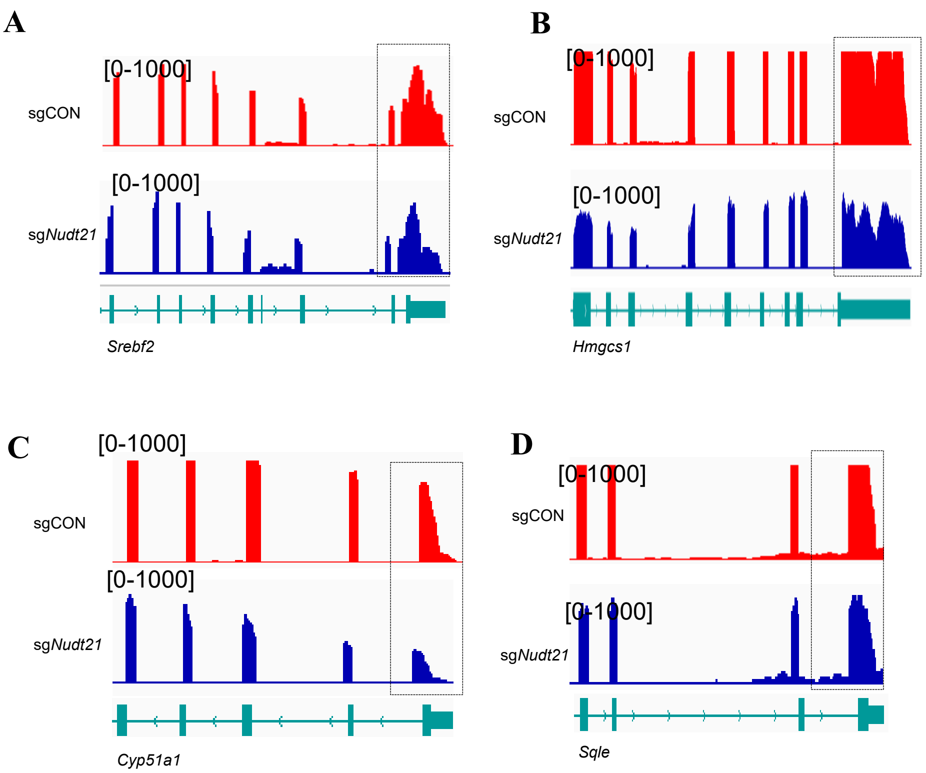

Figure S6. **Nudt21 deficiency had no effect on the 3' UTR length of cholesterol biosynthesis-related genes.**

(**A-D**) Read coverage plots of cholesterol synthesis-related genes (Srebf2, Hmgcs1, Cyp51a1, and Sqle) loci based on RNA-seq from sgCON (red) and sg*Nudt21*-(blue) MC38 cells.

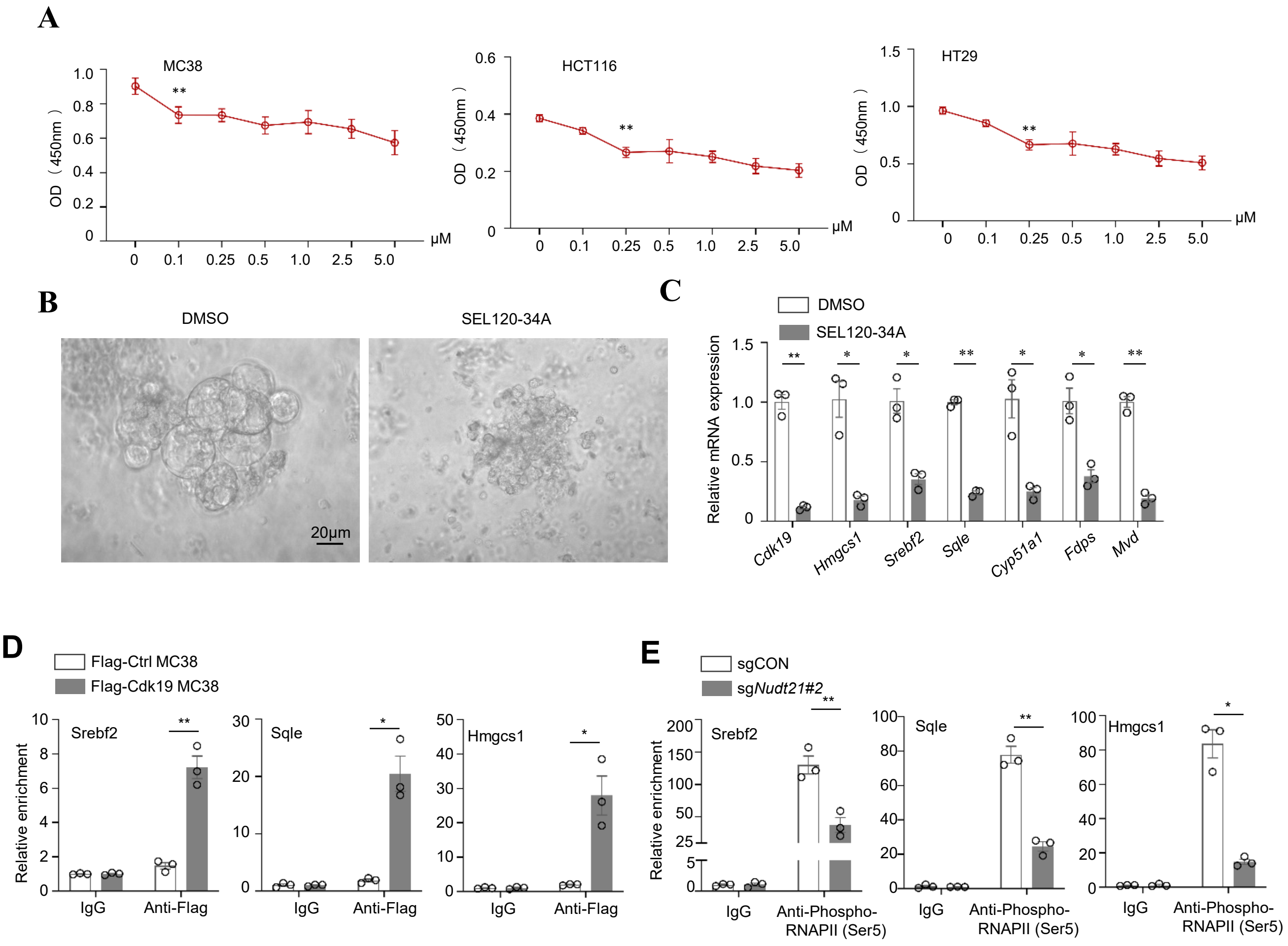

Figure S7. **CDK19 promotes CRC cell growth in vitro and directly acts on key cholesterol synthesis genes.**

(**A**) Cell vitality curve of HCT116, HT29, and MC38 cells treated with indicated concentrations of SEL120-34A, a CDK19 inhibitor, for 72 hours (n=5) .

(**B**) Representative images of patient-derived CRC organoid (PDO) treated with 1 μM SEL120-34A for 72 hours.

(**C**) RT-qPCR analysis of mRNA abundances of selected cholesterol biosynthesis-related genes in MC38 cells treated with DMSO or SEL120-34A (n=3).

(**D**) Flag CUT&RUN analysis of the promoter regions of *Srebf2, Sqle*, and *Hmgcs1* in MC38 cells expressing Flag-Cdk19 or vector control (n=3).

(**E**) Phospho-RNAPII (Ser5) CUT&RUN-QPCR analysis of the promoter regions of *Srebf2, Sqle*, and *Hmgcs1* in sgCON and sg*Nudt21*-MC38 cells (n=3).

Data represent the mean ± SEM. *p < 0.05, **p < 0.01 by Welch's test (**C**, **D**, and **E**) and two-way ANOVA (**A**).

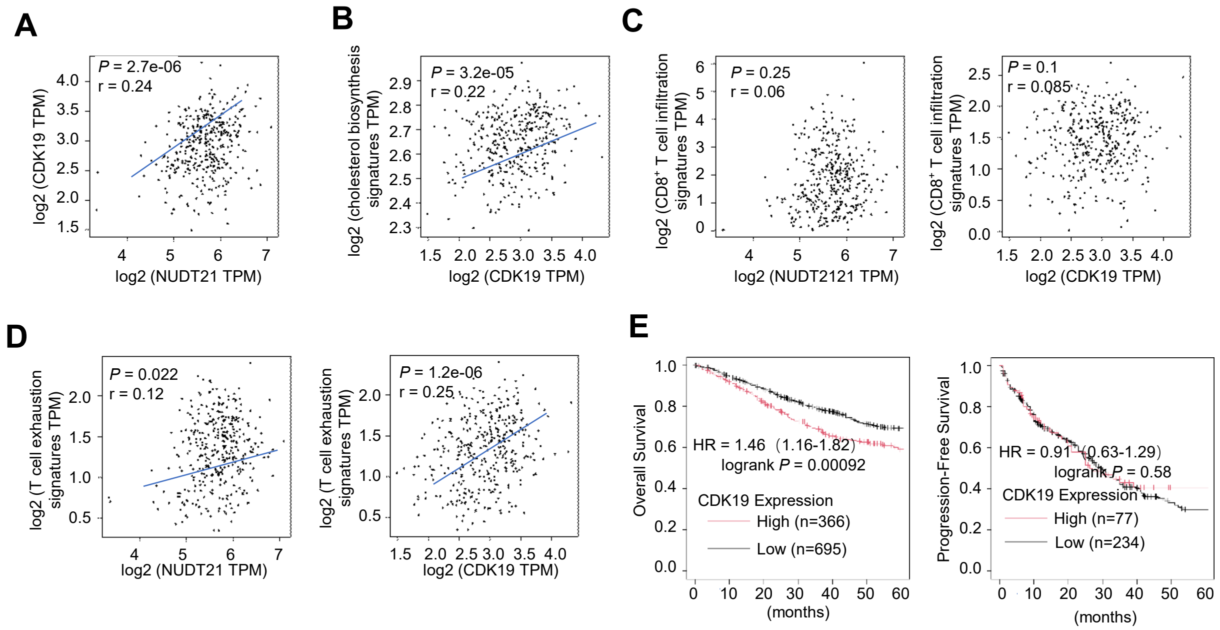

Figure S8. **Association between NUDT21 and CDK19 expression levels and clinical or molecular features in TCGA-COAD and -READ database.**

(A) Correlation between NUDT21 and CDK19 expression in TCGA-COAD and –READ database (n=367).

(B) Correlation between CDK19 expression and the score of cholesterol biosynthesis-related gene signature in TCGA-COAD and -READ database (n=367).

(C) Correlation between NUDT21 expression and the score of T cell infiltration-related gene signature (CD8a and CD8b) in TCGA-COAD and -READ database (n=367).

(D)) Correlation between CDK19 expression and the score of T cell exhaustion-related gene signature (*HAVCR2, TIGIT, LAG3*, *PDCD1, LAYN, CTLA4*, and *LAIR1*) in TCGA-COAD and -READ database (n=367).

(E) Kaplan-Meier plots of overall survival (n=1061) **(A)** and progression-free survival (n=311) **(B)** in patients from CRC datasets according to NUDT21 expression levels.

Pearson correlation coefficient (r) and p value are marked **(A, B, C** and **D)**. p values were calculated by Log-rank (Mantel-Cox) test **(E)**.
