# Supplementary material for: NUDT21‐mediated Alternative Polyadenylation of CDK19 Reprograms Cholesterol Biosynthesis to Drive Colorectal Cancer Progression
Source: Adv Sci (Weinh). 2025 Nov 19;13(7):e18346. doi: 10.1002/advs.202518346 (PMC12866688; doi:10.1002/advs.202518346)
Supplement: Supplementary file 3 — Supplemental Table 2 [file ADVS-13-e18346-s001.docx]

**Supplementary Table S2. Primer sequences used in the study.**

The target sequences of sgRNA used for genome editing.

| Target | Sequence |
| --- | --- |
| mouse-sg*Nudt21* | F1: AAGTTGTCCCCAGCTGCAGC  R1: GCTGCAGCTGGGGACAACTT |
|  | F2: CAGATACTTGGTCGTCAAGA  R2: TCTTGACGACCAAGTATCTG |
| human-sgNudt21 | F1: CCAGCCGGTCTGCGAGCGAT  R1: ATCGCTCGCAGACCGGCTGG |
|  | F2: TTCTGATTGTACATGAGCAC  R2: GTGCTCATGTACAATCAGAA |
| mouse-sg*Cdk19* | F1: AACTCCACTCTCTGTACAGC  R1: GCTGTACAGAGAGTGGAGTT |
|  | F2: CGCGTGGTCTCTTCAGAACT  R2: AGTTCTGAAGAGACCACGCG |

The primers used for PCR.

| Target | Sequence |
| --- | --- |
| mouse-*Hmgcs1* | F: AAGTTGTCCCCAGCTGCAGC  R: GCTGCAGCTGGGGACAACTT |
| mouse-*mvd* | F: ATGGCCTCAGAAAAGCCTCAG  R: TGGTCGTTTTTAGCTGGTCCT |
| mouse-*Sqle* | F: CCAGCCGGTCTGCGAGCGAT  R: ATCGCTCGCAGACCGGCTGG |
| mouse-*Cyp51* | F: TTCTGATTGTACATGAGCAC  R: GTGCTCATGTACAATCAGAA |
| mouse-*Fdps* | F: GGAGGTCCTAGAGTACAATGCC  R: AAGCCTGGAGCAGTTCTACAC |
| mouse-*Srebf2* | F: GCAGCAACGGGACCATTCT  R: CCCCATGACTAAGTCCTTCAACT |
| mouse-*Cdk19* CDS | F: GAGTCAAAATAGCTGACATGGGT  R: CCGGAGCCCGATACCAAAA |
| mouse-*Cdk19 long 3’*UTR | F: GATAGAGGCGGAAGCAAT  R: CAAGAGAACGAGGAATGAAG |
| mouse-*Ccl5* | F: GCTGCTTTGCCTACCTCTCC  R: TCGAGTGACAAACACGACTGC |
| mouse-*Cxcl10* | F: CCAAGTGCTGCCGTCATTTTC  R: GGCTCGCAGGGATGATTTCAA |
| mouse-*Ccl2* | F: TTAAAAACCTGGATCGGAACCAA  R: GCATTAGCTTCAGATTTACGGGT |
| mouse-*Cxcl16* | F: CCTTGTCTCTTGCGTTCTTCC  R: TCCAAAGTACCCTGCGGTATC |
| mouse-*Ccl25* | F: TTACCAGCACAGGATCAAATGG  R: CGGAAGTAGAATCTCACAGCAC |
| mouse-*β-actin* | F: ACTGCCGCATCCTCTTCCTC  R: AACCGCTCGTTGCCAATAGTG |
| human-Cdk19 CDS | F: ATATCCATGTCGGCTTGTAGAGA  R: GCTTTTGATGCACGGTGAAAC |
| human-Cdk19 long 3’UTR | F: GTGTGCTGCCTCATTCCTGGT  R: TCCTTCAAGCCTCTGTTGGTCT |
| human-β-actin | F: GGTTCCGCTGCCCTGAGG  R: GGAGTTGAAGGTAGTTTCGTGGATG |
| mouse-*Cdk19* long 3’UTR knockout genotyping primers | F: TAGAAGCTCTGTTCTTGCCCTC  R1: TCCATTATGTGTCTGTTCCT  R2: CTTCGCTTCCTTCACAGAG |
| mouseC-*Srebf2* | F: AAGCACCGCACTGAACCATCT  R: ACACAAGACAGACTAGCCAAGGAA |
| mouseC-*Sqle* | F: ACTGGTCTGGAACACGCTATGC  R: AGAGAAGTCAGTAGGTGGAGATGGA |
| mouseC-Hmgcs1 | F: GGTCGGTGGCTATAAAGCTG  R: CGGGACACTCACCCAAAG |
| Non-target gene | F: ATGCCTAACTTCCAGTTCCAGG  R: AGCTTAGAGCAGAAAGCTGGT |
